# Inner Co Synergizing Outer Ru Supported on Carbon Nanotubes for Efficient pH-Universal Hydrogen Evolution Catalysis

**DOI:** 10.1007/s40820-022-00933-2

**Published:** 2022-09-14

**Authors:** Jian Chen, Yuan Ha, Ruirui Wang, Yanxia Liu, Hongbin Xu, Bin Shang, Renbing Wu, Hongge Pan

**Affiliations:** 1 Institute of Science and Technology for New Energy, Xi’an Technological University, Xi’an, 710021 People’s Republic of China; 2 School of Advanced Materials and Nanotechnology, Xidian University, Xi’an, 710126 People’s Republic of China; 3 Department of Materials Science, Fudan University, Shanghai, 200433 People’s Republic of China; 4 State Key Laboratory of New Textile Materials and Advanced Processing Technologies, Wuhan Textile University, Wuhan, 430073 People’s Republic of China; 5 State Key Laboratory of Silicon Materials and School of Materials Science and Engineering, Zhejiang University, Hangzhou, 310027 People’s Republic of China

**Keywords:** Cobalt nanoparticles, Carbon nanotubes, Ru nanoclusters, Hydrogen evolution reaction

## Abstract

Exploring highly active but inexpensive electrocatalysts for the hydrogen evolution reaction (HER) is of critical importance for hydrogen production from electrochemical water splitting. Herein, we report a multicomponent catalyst with exceptional activity and durability for HER, in which cobalt nanoparticles were in-situ confined inside bamboo-like carbon nanotubes (CNTs) while ultralow ruthenium loading (~ 2.6 µg per electrode area ~ cm^−2^) is uniformly deposited on their exterior walls (Co@CNTsǀRu). The atomic-scale structural investigations and theoretical calculations indicate that the confined inner Co and loaded outer Ru would induce charge redistribution and a synergistic electron coupling, not only optimizing the adsorption energy of H intermediates (Δ*G*_H*_) but also facilitating the electron/mass transfer. The as-developed Co@CNTsǀRu composite catalyst requires overpotentials of only 10, 32, and 63 mV to afford a current density of 10 mA cm^−2^ in alkaline, acidic and neutral media, respectively, representing top-level catalytic activity among all reported HER catalysts. The current work may open a new insight into the rational design of carbon-supported metal catalysts for practical applications.

**Supplementary Information:**

The online version contains supplementary material available at 10.1007/s40820-022-00933-2.

## Highlights

- A multicomponent Co@CNTs|Ru catalyst has been rationally designed, in which Co nanoparticles are *in-situ* confined inside CNTs while trace Ru loading is uniformly deposited on their exterior walls.
- Co and Ru nanoparticles spatially confined by the inner and outer surface of CNTs, respectively, would induce charge redistribution and a synergistic electron coupling.
- Co@CNTs|Ru catalyst exhibits an unprecedented hydrogen evolution reaction (HER) activity in all pH-range, representing a new record among all the previously reported HER catalysts.

**Supplementary Information:**

The online version contains supplementary material available at 10.1007/s40820-022-00933-2.

## Graphical abstract

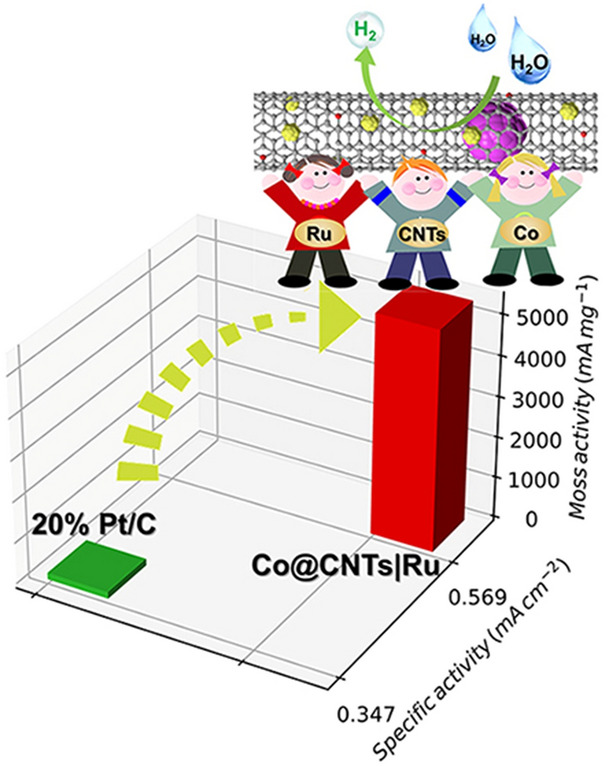

## Introduction

Hydrogen (H_2_) is regarded as an ideal alternative to traditional fossil fuels due to its high gravimetric energy density, earth-abundance and carbon-free emission [1–6]. Electrochemical water splitting provides a green and sustainable way for high-purity hydrogen production, especially when it is coupled with intermittent energy, such as solar and wind power [7–9]. Nevertheless, this way heavily depends on cathodic hydrogen evolution reaction (HER) catalysts that can effectively reduce the overpotential caused by the sluggish proton-coupled electron transfer kinetics. Platinum (Pt)-based materials have been widely considered as the most active HER catalyst with a minimum overpotential owing to its favorable hydrogen binding energy [10, 11]. Unfortunately, the scarcity and high price of Pt seriously restricted its large-scale application [12, 13]. In this regard, it is of significant importance to explore alternatives to Pt-based catalysts without sacrificing the HER activity based on earth-abundant elements. In addition, considering HER is employed in different applications such as proton exchange membrane water electrolysis in acidic solution, microbial electrolysis cells under neutral conditions and alkaline water electrocatalysis, it is highly demanded to explore catalysts that can operate and withstand well in a pH-universal electrolysis system.

Among various candidates, 3*d* transition metal (TM) coupled with carbon composites, especially with TM encapsulated by carbon nanotubes (TM@CNTs) have been demonstrated to be competitive HER catalysts. Within this composite, CNTs not only prevent the acid/alkaline corrosion of TM but also provide a spatial nanoconfinement for the facilitated electron/charge transfer channel and may serve as active sites for HER via a TM-modulated electron spin density and charge redistribution. For example, Yan et al. synthesized a hybrid composite consisting of the encapsulation of Ni nanoparticles into N-doped carbon nanotube/nanorod and found that it displayed a good HER performance with an overpotential of 134 mV to drive a current density of 10 mA cm^−2^ and an impressive long-term durability in 0.1 M KOH [14]. Yu et al. employed N-CNTs-encapsulated Fe nanoparticles on iron foam as an integrated electrode for HER and demonstrated that it required an overpotential of 525 mV to afford a current density of 10 mA cm^−2^ in neutral media [15]. Chen et al. constructed a composite by embedding Co nanoparticles into N-doped CNTs grafted onto reduced graphene oxide, which could achieve overpotentials of 108 and 87 mV at 10 mA cm^−2^ in 1.0 M KOH and 0.5 M H_2_SO_4_, respectively [16]. Cao et al. prepared N-CNTs arrays with co-implanted Co_4_N nanoparticles and single-atom Co and verified that the synergistic effects among components could effectively tailor the d-band center for facilitated electron transfer, enabling a robust HER performance with overpotentials of 78 and 86 mV at 10 mA cm^−2^ in 0.5 M H_2_SO_4_ and 1.0 M KOH, respectively [17]. Despite prominent advances that have been made, the optimization of intrinsic hydrogen binding energy of TM@N-CNTs composites is limited by the available strategies and thus their actual HER performance is still inferior to that of Pt-based catalyst, especially in the view of the pH-universal range.

Herein, we have innovatively adopted a lysozyme (Lys)-assisted zeolitic imidazole framework (ZIF)-driven strategy for the synthesis of a multicomponent catalyst with cobalt (Co) and ruthenium (Ru) nanoparticles confined by the inner and outer surface of N-CNTs, respectively (Co@CNTsǀRu). The introduction of Ru is mainly based on the consideration of its similar bond strength with hydrogen but the much lower price to Pt [18, 19]. Benefiting from the optimized adsorption energy of H intermediates caused by a synergistic electron coupling and the facilitated electron/mass transfer path, the Co@CNTsǀRu composite catalyst exhibited an unprecedented HER activity in all pH-range, with overpotentials of only 10, 32, and 63 mV at 10 mA cm^−2^ in 1.0 M KOH, 0.5 M H_2_SO_4_ and 1.0 M phosphate-buffered saline (PBS) solution, respectively. This catalytic performance not only outperforms that of the state-of-the-art Pt/C catalyst but also may represent a new record among all the previously reported TM-based catalysts. Additionally, it is worth emphasizing that the loading amount of immobilized Ru onto the outer surface of N-CNTs is only 2.6 µg per electrode area ~ cm^−2^, which is significantly lower than those of all reported Ru-based HER catalyst in the form of ultrafine nanoparticles, alloys or single-atoms deposited onto the carbon support [20 − 23], further demonstrating a superiority in the view of materials cost.

## Experimental Section

### Synthesis of ZIF-67@Lys Precursor

In a typical synthesis of ZIF-67@Lys precursor, 10 g of Lysozyme was dissolved by 35 mL of an aqueous solution to form a clear solution, and then mixed with 5 mL of aqueous solution containing 148.7 mg of Co(NO_3_)_2_·6H_2_O. Next, 10 mL of an aqueous solution containing 3.284 g of 2-methylimidazole (2-MeIm) was further injected into the above solution and stirred for 24 h at ambient temperature. Finally, the ZIF-67@Lys precursor was obtained by centrifugation and drying at 60 °C.

### Synthesis of Co@CNTs Catalyst

ZIF-67@Lys precursor was pyrolyzed in a tube furnace using a two-stage programmed heating process, i.e., at 550 °C for 3 h under N_2_ atmosphere with a heating rate of 2 °C min^–1^ and then at 900 °C for another 3 h in N_2_ with a heating rate of 3 °C min^–1^. The Co@CNTs catalyst was obtained when the tube furnace was cooled to ambient temperature naturally.

### Synthesis of Co@CNTs|Ru Catalyst

Co@CNTs (50 mg) and ruthenium chloride (RuCl_3_, 5 mg) were dispersed in ethanol. The mixture was agitated in a sonication bath for 30 min and further stirred overnight using a magnetic stirrer. Then, the mixtures were obtained by centrifugation and drying at 60 °C. Finally, the mixtures were directly annealed at 700 °C under N_2_ atmosphere for 3 h to obtain Co@CNTs|Ru catalyst. As a comparison, Co@CNTs|Ru catalysts with different Ru contents were also prepared by using the same method. Here, the obtained composite catalysts were named as Co@CNTsǀRu-*n*, where *n* means the amount of added RuCl_3_ precursor. Note that Co@CNTsǀRu-5 mg is simplied as Co@CNTsǀRu.

### Materials Characterizations

The as-synthesized samples were studied by field-emission scanning electron microscope (FESEM, JEOL JSM-6700F), transmission electron microscope (TEM, JEOL JEM-2100F), aberration-corrected high-angle annular dark-field scanning transmission electron microscopy (HAADF-STEM), X-ray diffraction (XRD, Rigaku, Cu K*α* radiation) and X-ray photoelectron spectroscopy (XPS, Kratos XSAM-800, Mg Kα radiation source). The Brunauer–Emmett–Teller (BET) surface area characterizations were analyzed by nitrogen sorption measurement.

### Preparation of Working Electrodes

5 mg of samples and 30 µL of Dupont Nafion 117 solution (10 wt%) were added in ethanol (970 µL), followed by sonicated for 30 min to get a homogeneous suspension. Then, 10 μL of the catalyst ink was loaded onto a pre-polished glassy carbon electrode (D = 5.0 mm) of a rotating ring disk electrode (RRDE) (loading density: 0.25 mg cm^–2^). Note that ruthenium loading (1.04%) of Co@CNTs|Ru catalyst is ~ 2.6 µg per electrode area ~ cm^−2^, while the Pt loading of 20 wt% Pt/C catalyst is ~ 50 µg per electrode area ~ cm^−2^.

### Electrochemical Measurements

All the electrochemical measurements were carried out on a CHI 760E electrochemical workstation (CH Instruments, Inc., Shanghai) with a standard three-electrode system [24]. Electrochemical tests were performed on rotating disk electrodes. The Ag/AgCl electrode in saturated KCl and graphite rod were served as the reference and the counter electrode, respectively. The HER performance was evaluated in N_2_-saturated electrolytes with different pH values. The electrocatalytic activities of the samples were examined by obtaining polarization curves using linear sweep voltammetry (LSV) with a scan rate of 5 mV s^–1^ at room temperature. The stability measurements were performed by cyclic voltammetry scanning for 5000 cycles (sweep rate, 50 mV s^–1^) and time-dependent current density curves (*i*-*t* curves for 50 h at a current density of 10 mA cm^–2^). CV method was also used to determine the electrochemical double-layer capacitances (*C*_dl_) at non-faraday intervals. Electrochemically active surface area (ECSA) could be evaluated from the slope of the plot of the charging current versus the scan rate, which was directly proportional to *C*_dl_. The ECSA is the electrochemically active surface area, which can be calculated from Eq. (1):

1 $$\mathrm{ECSA}=\frac{{C}_{dl}}{{C}_{s}}=\frac{{C}_{dl} (mF {cm}^{-2})}{0.04 (mF {cm}^{-2})}$$

where *C*_s_ is the specific capacitance of 1 cm^2^ atomically smooth standard electrode (here *C*_s_ = 0.04 mF cm^–2^ in 1.0 M KOH solution) [25]. The ECSA of the Co@CNTs|Ru catalyst is calculated to be 1.59, which is larger than that of Pt (1.07), implying more exposure of active sites in the Co@CNTs|Ru catalyst.

The specific activity is calculated by Eq. (2):

2 $$\text{Specific\, activity}= \frac{\mathrm{j }(\mathrm{mA }{cm}^{-2})}{ECSA}$$

The mass activity is calculated by Eq. (3):

3 $$\text{Mass activity}= \frac{\mathrm{j }(\mathrm{mA }{cm}^{-2})}{metal\, loading\, (mg \,{cm}^{-2})}$$

where the *j* is current density from the LSV.

### TOF Calculation

The number (N_Ru_) of surface-active sites per cm^−2^ is calculated based on Eqs. (4) and (5):

4 $$n (\mathrm{mol})=\frac{{m}_{Ru}}{{M}_{Ru}}=\frac{{N}_{Ru}}{{N}_{A}}$$

5 $${N}_{Ru}=\frac{0.25\, mg\, {cm}^{-2}\times 1.04\mathrm{\%}}{101.1\times {10}^{3}\, mg\, {mol}^{-1}}\times 6.022\times {10}^{23}\frac{1}{mol}=1.548\times {10}^{16} \,site\, {cm}^{-2}$$

where m is the Ru loading of Co@CNTs|Ru on the per electrode area, M is molar mass of Ru (101.1 g mol^−1^), N_A_ is a constant (6.022 × 10^23^ mol^−1^).

Then the per-site TOF is calculated by Eq. (6) [26]:

6 $${TOF}_{per site}= \frac{the\, number\, of\, total \,hydrogen \,turn \,overs}{the \,number\, of \,surface\, active\, sites}$$

The number of total hydrogen is calculated from the current density using Eq. (7):

7 $$\left(j\frac{mA}{{cm}^{2}}\right)\left(\frac{1 A}{1000\, mA}\right)\frac{1C/s}{1\, A}\left(\frac{1 \,mol\, {e}^{-}}{96485.3\, C}\right)\left(\frac{1\, mol\, {H}_{2}}{2\, mol\, {e}^{-} }\right)\left(\frac{6.022 \times {10}^{23}molecules\, {H}_{2}}{1 \,mol \,{H}_{2}}\right)=3.12 \times {10}^{15} \frac{{H}_{2}/s}{{cm}^{2}} per \frac{mA}{{cm}^{2}}$$

The current density at overpotential of 100 mV in 1.0 M KOH is *j* mA cm^−2^; therefore, the TOF per site is calculated as Eq. (8)

8 $$\frac{\left(\times {10}^{15} \frac{{H}_{2}/s}{{cm}^{2}} per \frac{mA}{{cm}^{2}}\right)\left(j\frac{mA}{{cm}^{2}}\right)}{\left(1.548 \times {10}^{16}\right)}=0.20j \frac{{H}_{2}/s}{\text{surface site}}$$

### DFT Calculation

The first-principle DFT calculations were performed by Vienna Ab initio Simulation Package (VASP) with the projector augmented wave (PAW) method [27]. The exchange-functional was treated using the generalized gradient approximation (GGA) of Perdew-Burke-Ernzerhof (PBE) functional [28]. The energy cutoff for the plane wave basis expansion was set to 450 eV and the force on each atom less than 0.03 eV Å^−1^ was set for convergence criterion of geometry relaxation. A 15 Å vacuum was added along the z direction in order to avoid the interaction between periodic structures. The Brillouin zone integration is performed using 2 × 2 × 1 k-point sampling. The self-consistent calculations apply a convergence energy threshold of 10^−4^ eV. The DFT-D3 method was employed to consider the van der Waals interaction [29]. The Free energies of the adsorption atomic hydrogen (Δ*G*_H_) is calculated by Eq. (9) [30]:

9 $$\Delta {\mathrm{G}}_{H}={\Delta E}_{DFT}+{\Delta E}_{ZPE}-\mathrm{T\Delta S}$$

where ${\Delta E}_{DFT}$ is the DFT energy difference and the ${\Delta E}_{ZPE}$ and the $\mathrm{T\Delta S}$ terms are obtained based on vibration analysis.

## Results and Discussion

### Materials Characterization

**Fig. 1:**
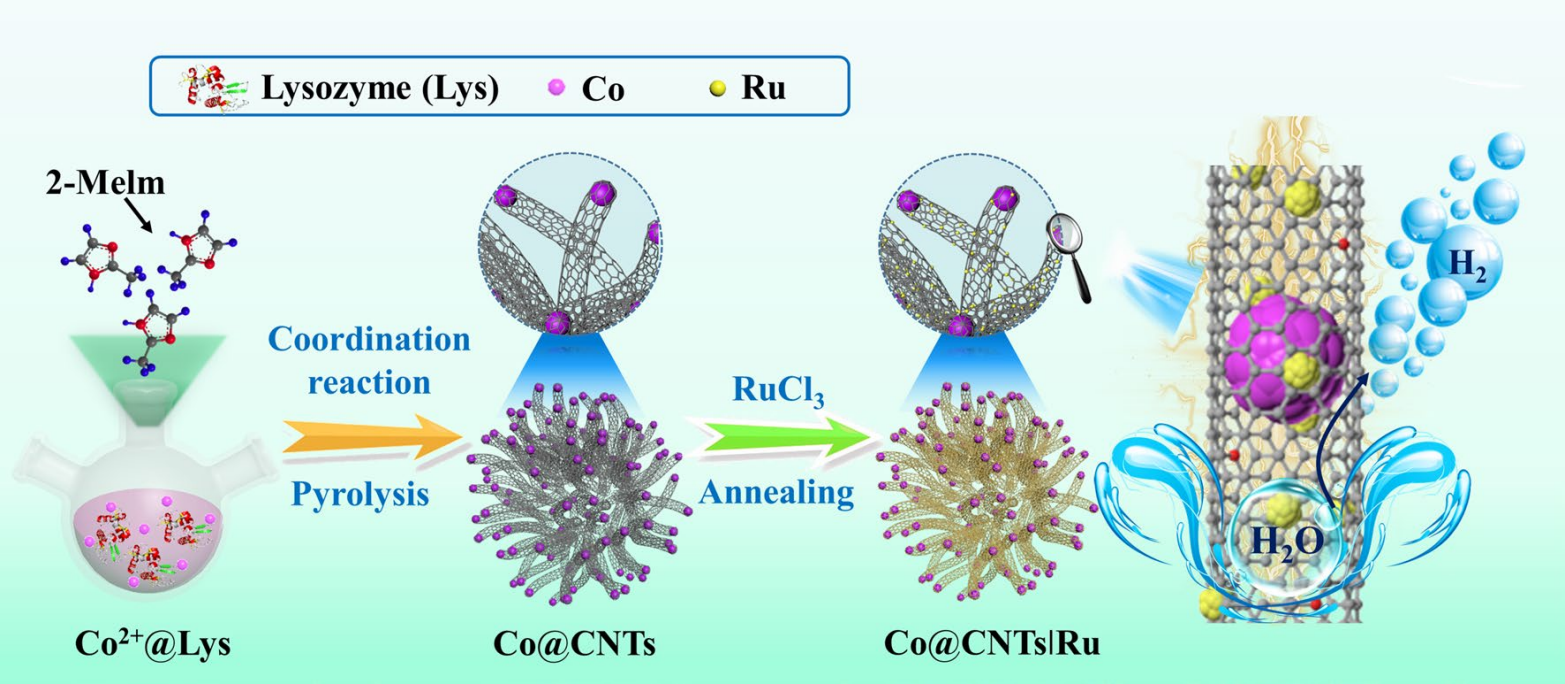
Synthetic procedure for the preparation of Co@CNTs|Ru composites

The synthetic procedure of the multicomponent Co@CNTsǀRu catalyst is schematically illustrated in Fig. 1. Firstly, Co-based zeolitic imidazolate frameworks (ZIF-67) surrounded by lysozyme (ZIF-67@Lys) precursor was synthesized via the coordination of Co^2+^ with 2-methylimidazole in the presence of lysozyme in an aqueous solution at room temperature. After a pyrolysis process, the liberated Co^2+^ from the precursor were reduced to Co nanoparticles, while the coordinated organic ligands together with the surrounded Lys were completely in-situ carbonized into bamboo-like CNTs, generating a composite consisting of Co nanoparticles confined into the inner surface of CNTs (Co@CNTs). Lastly, with the thermal reduction of RuCl_3_-treated Co@CNTs, ultrafine Ru nanoclusters were uniformly anchored onto the surface of CNTs, leading to the final multicomponent Co@CNTsǀRu catalyst. To understand the synergistic effect of the components, Co@CNTsǀRu composite with different Ru loadings, Co@CNTs composite, bare CNTs after removing Co via acid leaching, as well as the Ru nanoclusters anchored on bare CNTs samples (CNTsǀRu) were also prepared for comparison.

**Fig. 2:**
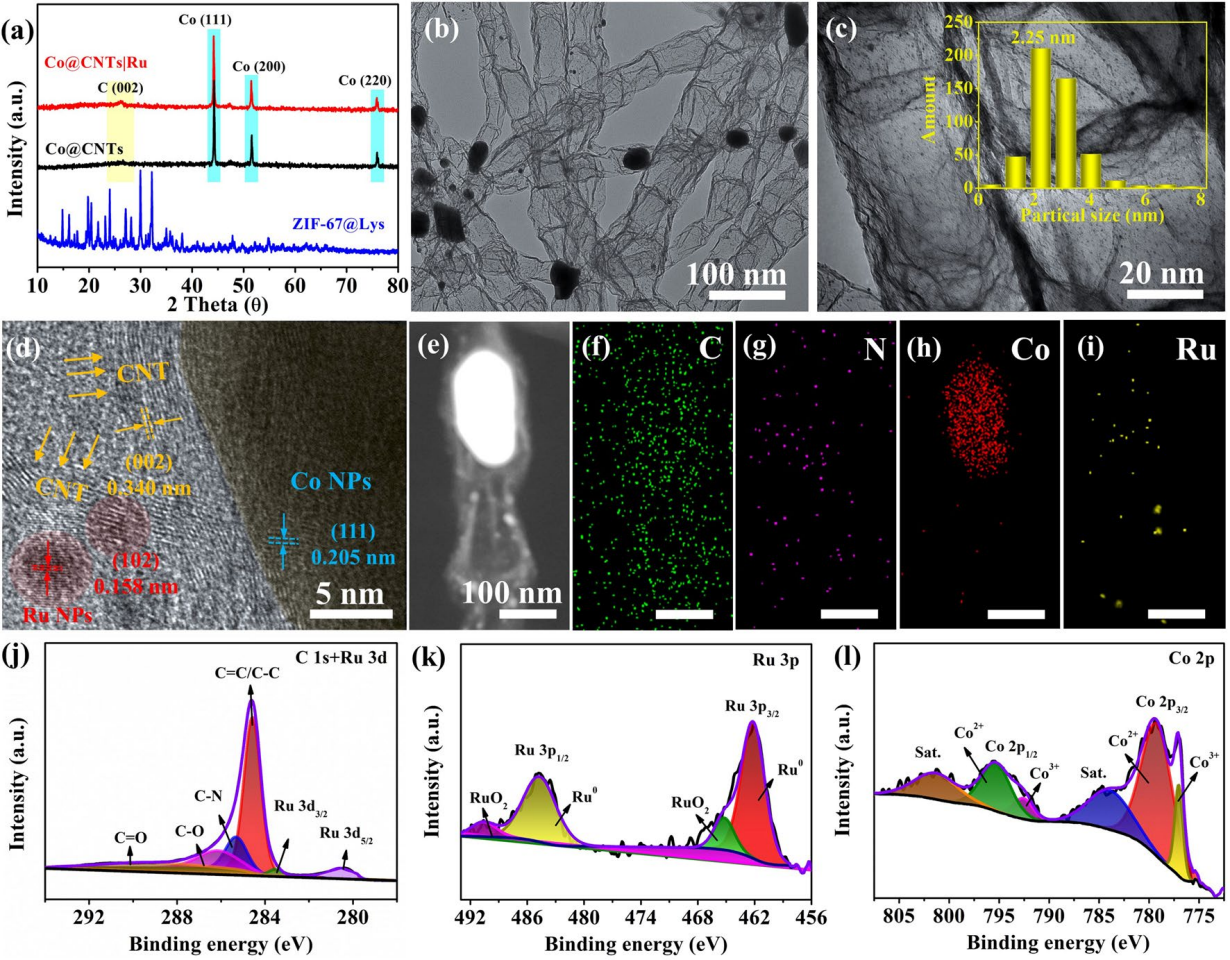
**a** XRD patterns of ZIF-67@Lys precursor, Co@CNTs and Co@CNTs|Ru composites; **b, c** TEM images (Inset c: the particle size distribution of Ru nanoclusters), and **d** HRTEM image of Co@CNTs|Ru composites; **e − i** HAADF-STEM images and corresponding EDX elemental mappings of Co@CNTs|Ru composites; **j − l** high-resolution XPS spectrum of C 1*s*, Ru 3*p* and Co 2*p* of Co@CNTs|Ru composites

FESEM and TEM were performed to study the morphology and microstructure of the as-prepared products. As shown in Fig. S1, the as-synthesized ZIF-67@Lys precursor was monodisperse and had a dodecahedron morphology with a relatively smooth surface, suggesting that the introduction of Lysozyme did not change the crystalline structure of the ZIF-67. Figure 2a presents the XRD pattern of the pyrolyzed products. In addition to one peak belongs to graphitic carbon, three peaks at 44.7°, 52°, and 76° corresponding to (111), (200), and (220) facets of Co, respectively, could be identified, suggesting the complete conversion from the ZIF-67@Lys precursor to Co@CNTs composites. Figure S2a-c displayed the panoramic FESEM images of the representative Co@CNTs composites, in which a high density of CNTs with a length up to tens of micrometers was clearly observed. TEM images further manifested that the composites consisted of Co nanoparticles encapsulated by the bamboo-like CNTs with a diameter ranging from 50 to 80 nm (Fig. S2d-f). Each CNT confined one Co nanoparticle only at the top, implying Co-catalyzed growth mechanism for the generation of CNTs [31 − 33]. Note that the introduction of Lys was favorable to the formation and the growth of bamboo-like N-doped CNTs (Fig. S3).

After a thermal reduction of RuCl_3_-treated Co@CNTs, no obvious change was observed in the XRD patterns of the obtained product (Fig. 2a). Nevertheless, TEM image clearly illustrated that many Ru nanoclusters with an average size of 2.25 nm were generated and uniformly anchored onto the surface of bamboo-like CNTs (Fig. 2b-c). High-resolution TEM (HRTEM) image further revealed that the Co nanoparticle was tightly confined into the inner surface of CNTs while the Ru nanoclusters were embedded within their outer surface. The distinct lattice fringes with *d*-spacings of 0.205, 0.340, and 0.158 nm were indexed to the (111), (002), and (102) lattice planes of the metallic Co, graphitic carbon and metallic Ru phases, respectively (Fig. 2d). Interestingly, no direct contact between Co nanoparticles and Ru nanoclusters was observed in the Co@CNTsǀRu composite, possibly be due to the spatial separation of CNTs wall. Such multicomponent composite catalyst with unique spatial confinement enabled by CNT could not only greatly improve charge transfer ability and ensure more exposure of active sites but also is favorable to charge redistribution and a synergistic electron coupling. A high-angle annular dark-field scanning TEM (HAADF-STEM) image of representative Co@CNTsǀRu composite further confirmed that the Co nanoparticle and Ru nanocluster were separated by the inner and outer wall of CNTs. The corresponding elemental mappings analysis proclaimed the coexistence of Co, C, N, and Ru elements within the composites (Figs. 2e-i and S4).

Figure S5 shows XRD patterns of Co@CNTsǀRu composites with different Ru loadings. When the amount of added RuCl_3_ was less than 10 mg, in addition to Co and graphitic carbon phases, no diffraction peaks of Ru were observed, suggesting that the added RuCl_3_ less than 10 mg had no obvious influence on the phase structure of the Co@CNTs. In comparison with diffraction peaks of Co@CNTs, the peaks of Co@CNTs|Ru-5 mg and Co@CNTs|Ru-10 mg slightly shifted to lower angles, suggesting that the formed Ru nanoparticles with smaller size dispersed well in Co@CNTs [18]. With increasing the amount of RuCl_3_ to 20 mg and even to 40 mg, new peaks centered at 37.8°, 42.4°, 44.8°, 58°, 69.7°, and 78.8° were observed, which could be indexed to the (100), (002), (101), (102), (110), and (103) planes of Ru nanoparticles (JCPDS No. 06–0663). TEM images of Co@CNTs|Ru with different Ru contents indicated that the particle size of Ru increased gradually with the increase of added RuCl_3_ content (Fig. S6).

XPS characterizations were further employed to investigate the elemental composition and surface chemical states. The XPS survey spectrum of Co@CNTsǀRu shows the existence of C 1*s*, N 1*s* Co 2*p*, and Ru 3*d* peaks (Fig. S7a). Further investigation of the high-resolution C 1*s* (Fig. 2j) and N 1*s* spectra (Fig. S7b) indicated the successful doping of N into the CNTs [34]. The high-resolution N 1*s* spectrum of Co@CNTs|Ru could be deconvoluted into four peaks, i.e., pyridinic-N (397.8 eV), Co–N and Ru–N (398.9 eV), pyrrolic-N (400.3 eV), and graphitic-N (401.4 eV), respectively, all of which were beneficial to the HER processes [14 − 16]. Specifically, the N-doping would rebalance the charge density of the Co@C system, leading to a tunable absorption ability of Co@C model to hydrogen [16]. Note that the signal peak of C 1*s* is partially coincide with the signal peak of Ru 3*d*. According to Fig. 2j, the peak at around 280.0 and 284.0 eV may be related to the Ru^0^ species in the Co@CNTsǀRu composite, corresponding to Ru 3*d*_5/2_ and Ru 3*d*_3/2_, respectively [35]. The high-resolution Ru 3*p* spectrum exhibited two spin–orbit splitting of Ru 3*p*_3/2_ and Ru 3*p*_1/2_ (Fig. 2k), suggesting a strong coupling effect of Ru to the Co@CNTs [35]. The two peaks located at 484.6 and 462.0 eV were the characteristic features of metallic Ru while another two peaks centered at 491.9 and 466.1 eV were related to the RuO_2_. The presence of RuO_2_ might be caused by a slight oxidation of the sample when exposed to air [36]. The valence distribution of Co was further analyzed from the deconvolution peak of Co 2*p*. The spectrum showed two sets of spin–orbit doublets, corresponding to Co 2*p*_3/2_ and Co 2*p*_1/2_ with the shakeup satellite peaks (Fig. 2l). The Ru and Co contents of the composite from the XPS spectrum were approximately 0.97 and 21 wt%, respectively (Table S1). The ultralow loading of Ru was further verified by the inductively coupled plasma optical emission spectrometry (ICP) results. As shown in Table S1, the Ru contents in the Co@CNTsǀRu composites was as low as 1.04 wt%, suggesting the trace Ru in the composites with good balance of their cost. X-ray absorption spectroscopy (XAS) was employed to further investigate the electron configurations and local structures components. As shown in Fig. S8a, the X-ray near-edge structure spectroscopy (XANES) spectra indicated that the Co K-edge absorption energies for Co@CNTs and Co@CNTsǀRu were lower than those of CoO but close to a Co reference foil, suggesting that the valence state of Co in Co@CNTs and Co@CNTsǀRu would be Co^0^ [37]. The Co K-edge extended X-ray absorption fine structure (EXAFS) curves for Co@CNTs and Co@CNTsǀRu are shown in Fig. S8b. Compared to Co foil and Co@CNTs, an obvious shift of Co–Co bond for Co@CNTsǀRu was observed, further suggesting that the electron configurations and local structures of Co would change after the introduction of Ru.

The specific surface area and pore size distribution of Co@CNTsǀRu composite were studied by N_2_ adsorption/desorption isotherms. As shown in Fig. S9, the composites exhibited a large Brunauer–Emmett–Teller (BET) specific surface area of 143.18 m^2^ g^−1^ and had a both micropores and mesopores with the main pore diameter around 10 nm. Such a large BET surface area together with a hierarchical pore is believed to be favorable to exposing more abundant catalytic active sites and enhancing the rapid mass/charge transportation during the electrochemical reactions [38].

### Electrocatalytic Performance

**Fig. 3:**
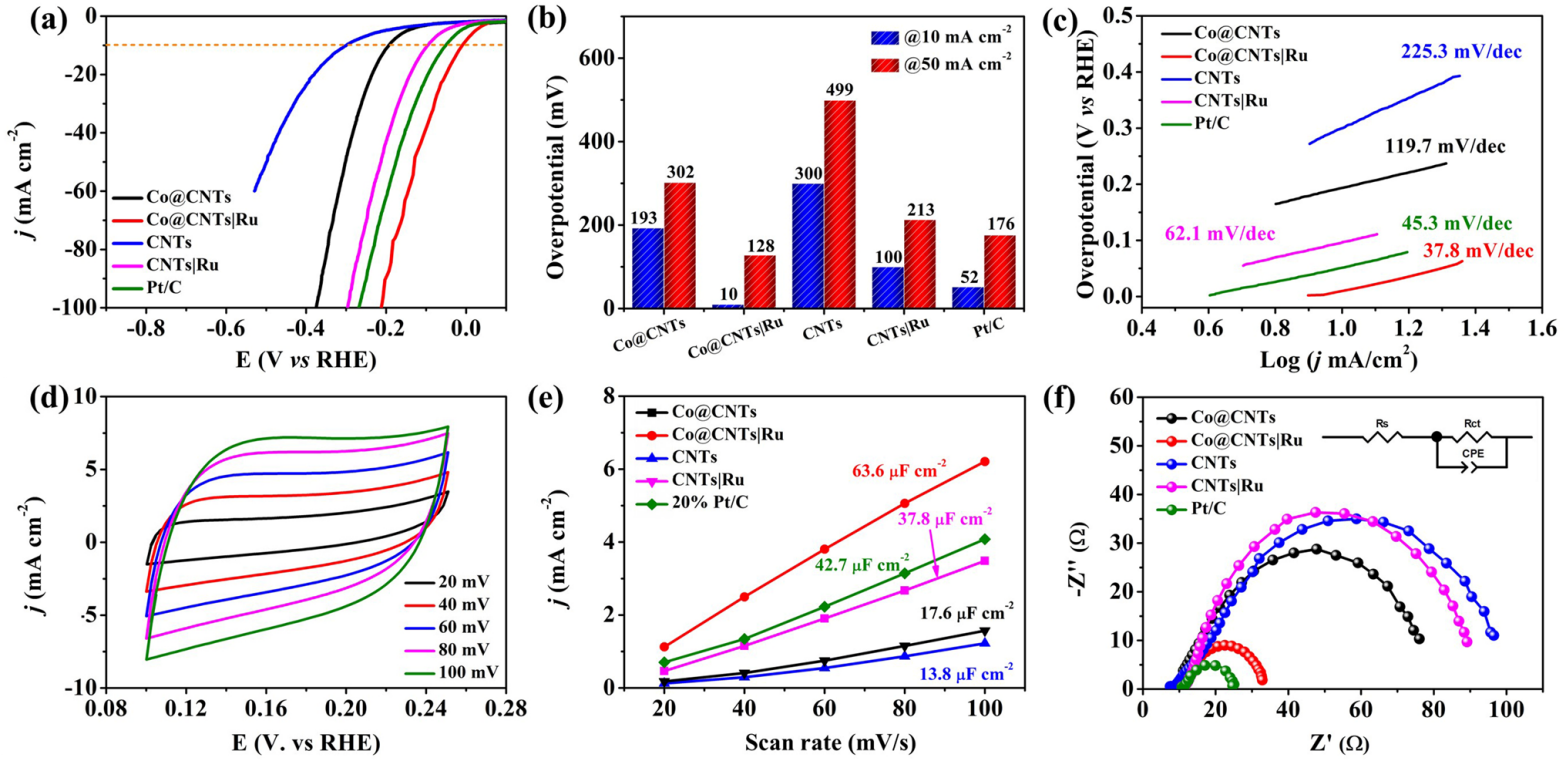
**a** LSV curves, **b** overpotentials at a current density of 10 mA cm^−2^, **c** Tafel plots of Co@CNTs, Co@CNTs|Ru, CNTs, CNTs|Ru and 20% Pt/C electrode; **d** CV curves for Co@CNTs|Ru electrocatalysts at different scan rates of 20, 40, 60, 80, and 100 mV s.^−1^; **e** *C*_dl_ values and **f** corresponding EIS spectra of Co@CNTs, Co@CNTs|Ru, CNTs, CNTs|Ru and 20% Pt/C electrode in 1.0 M KOH solution (inset: the equivalent circuit for EIS)

The electrocatalytic performance of the as-obtained Co@CNTsǀRu composite, Co@CNTs, CNTs, CNTs|Ru and commercial 20 wt% Pt/C for HER was evaluated using a typical three-electrode cell. Before comparison, the effect of Ru content on the catalytic performance of the composite was investigated. As shown in Fig. S10, LSV curves of Co@CNTs|Ru with different Ru contents suggested that increasing the content of Ru had a limited effect on the catalytic activity of composite catalyst. As a result, considering the materials cost, the Co@CNTs|Ru composite obtained by using 5 mg of RuCl_3_ was chosen a target sample. Figure 3a presents the polarization curves of different electrocatalysts received with linear sweep voltammetry (LSV) at a scan rate of 5 mV s^−1^ in 1.0 M KOH solution. All the polarization curves of samples were collected without *iR* correction. Strikingly, the Co@CNTsǀRu required an overpotential only 10 mV to achieve a current density of 10 mA cm^−2^, which was much lower than those of Co@CNTs (193 mV), CNTs (300 mV), CNTsǀRu (98 mV) and commercial 20% Pt/C (52 mV) (Fig. 3b). Furthermore, Co@CNTsǀRu could afford a large current density of 50 mA cm^–2^ at overpotential of only 128 mV, also surpassing Pt/C and most reported Ru-based catalysts (Fig. 3b and Table S2). As an effective criteria to estimate the HER kinetics within a certain potential range [39], the corresponding Tafel slopes of the above catalysts are shown in Fig. 3c. The Co@CNTsǀRu exhibited the smallest Tafel slope of Co@CNTs|Ru catalyst (37.8 mV dec^−1^) among the investigated samples, implying a facile kinetics and a Tafel–Volmer mechanism with the electrochemical desorption of H_2_ as the rate-determining step in the HER process [40]. Additionally, the electrochemical active surface area (ECSA) of the catalysts was also investigated. The ECSA is reflected from the double-layer capacitance (*C*_dl_), which can be obtained by deriving from the cyclic voltammetry (CV) curves *versus* the different scan rates under a non-faradaic potential range of 0.1 to 0.25 V *versus* RHE in 1.0 M KOH solution (Figs. 3d and S11). As shown in Fig. 3e, the Co@CNTs|Ru catalyst exhibited a *C*_dl_ value of 63.6 mF cm^−2^, which was the highest value among all of the studied catalysts (17.6 mF cm^−2^ for Co@CNTs, 13.8 mF cm^−2^ for CNTs, 37.8 mV cm^−2^ for CNTs|Ru and 42.7 mF cm^−2^ for commercial 20% Pt/C). The electrochemical impedance spectroscopy (EIS) fitting results showed that the charge transfer resistance of Co@CNTsǀRu was obviously smaller than those of other catalysts (Fig. 3f and Table S3). The above results showed that the CNTs with the co-existence of confined inner Co and loaded outer Ru would induce charge redistribution and a synergistic electron coupling, leading to more exposure of accessible active sites, and enhanced interfacial electron transport ability and thus an unprecedented electrocatalytic performance.

**Fig. 4:**
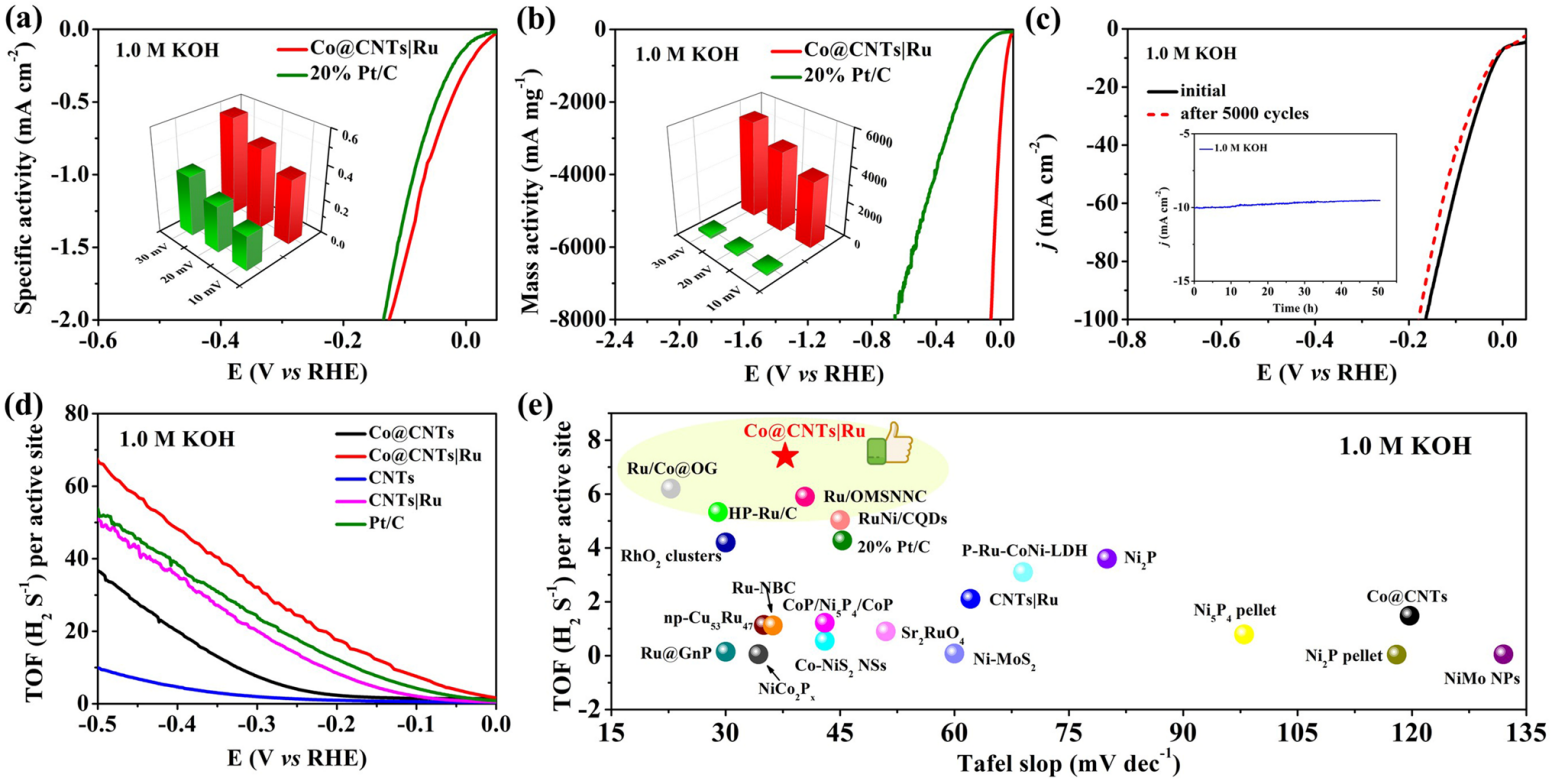
**a** Specific activities and **b** mass activities of Co@CNTs|Ru and Pt/C (Intet: specific activities (inset in **a**) and mass activities (inset in **b**) at different overpotentials of 10, 20, and 30 mV); **c** LSV curves for Co@CNTs|Ru before and after 5000 cycles of CV test (Inset: the i-t curves of Co@CNTs|Ru for 50 h); **d** TOF per surface metal site of Co@CNTs, Co@CNTs|Ru, CNTs, CNTs|Ru and Pt/C catalysts in 1.0 M KOH; **e** Comparison of TOF values and Tafel values of Co@CNTs|Ru, 20 wt% Pt/C catalyst and other recently reported HER electrocatalysts under the overpotential of 100 mV in 1.0 M KOH

For a fair comparison of catalytic activity, the polarization curves of Co@CNTs|Ru and Pt/C were further normalized by ECSA. The Co@CNTs|Ru demonstrated a higher specific activity than the Pt/C catalyst at the same potential from the ECSA-normalized LSV curves in 1.0 M KOH solution (Fig. 4a). Particularly, the specific activity of Co@CNTs|Ru (0.37 mA cm^−2^) showed ~ 1.85 times higher specific activity than that of Pt/C (0.20 mA cm^−2^) at an overpotential of 30 mV, implying a higher intrinsic per-site activity of the Co@CNTs|Ru catalyst [41]. Mass activity is closely related to the cost for practical applications. As shown in Fig. 4b, the mass activity of each catalyst was evaluated by normalizing the polarization curves with the mass of Ru (0.0026 mg per electrode area ~ cm^−2^) and Pt (0.05 mg per electrode area ~ cm^−2^). At the overpotential of 10 mV, the mass activity of Co@CNTs|Ru catalyst was 3706 mA mg^−1^, nearly 32 times that of commercial Pt/C (116 mA mg^−1^). Note that the specific and mass activities of Co@CNTs|Ru were also much higher than those of 10 wt% Ru/C catalyst (Fig. S12). Additionally, the Co@CNTs|Ru catalyst exhibited a high electrochemical stability in 1.0 M KOH. As shown in Fig. 4c, after 5000 cycles of scanning, the polarization curves of Co@CNTs|Ru was almost no change. The current–time (*i*–*t*) test (inset Fig. 4c) showed that the Co@CNTs|Ru catalyst only had less than 6.7% degradation of the initial current density after 50 h. The catalyst after long-durability test was investigated by XRD, XPS, and TEM characterizations. As shown in Fig. S13 (Supporting Information), XRD patterns of Co@CNTs|Ru catalyst before and after durability test were almost the same, confirming its good structural stability. TEM image of Co@CNTsǀRu catalsyt after the durability test showed that the Co and Ru nanoparticles still located inside the CNTs and on the outer suface of CNTs, reepectively (Fig. S14). The XPS spectra indicated no change in the valence states of Co (Fig. S15), further confirming that the Co@CNTsǀRu catalyst was stable. This excellent durability might be due to the strong affinity between Ru nanoparticles and Co@CNTs [42]. Therefore, it can be safely stated that Co@CNTs|Ru has significant advantages over Pt/C in terms of overall catalytic performance and cost.

Additionally, the turnover frequencies (TOF) values were also calculated to reveal the intrinsic activity of the catalyst. According to the estimated number of active sites, the TOF values of each active site of Co@CNTs, Co@CNTs|Ru, CNTs, CNTs|Ru and Pt/C in alkaline electrolyte was compared (Fig. 4d). The TOF values of Co@CNTs|Ru at η = 10, 100, 200 mV in KOH media were not only much higher than those of Co@CNTs, CNTs, CNTs|Ru and previously reported other contrast Co/Ru-based catalysts but also ~ 2 times that of the state-of-the-art 20% Pt/C (Fig. S16), revealing a higher intrinsic electrocatalytic activity of Co@CNTs|Ru. (Fig. 4e and Table S4).

**Fig. 5:**
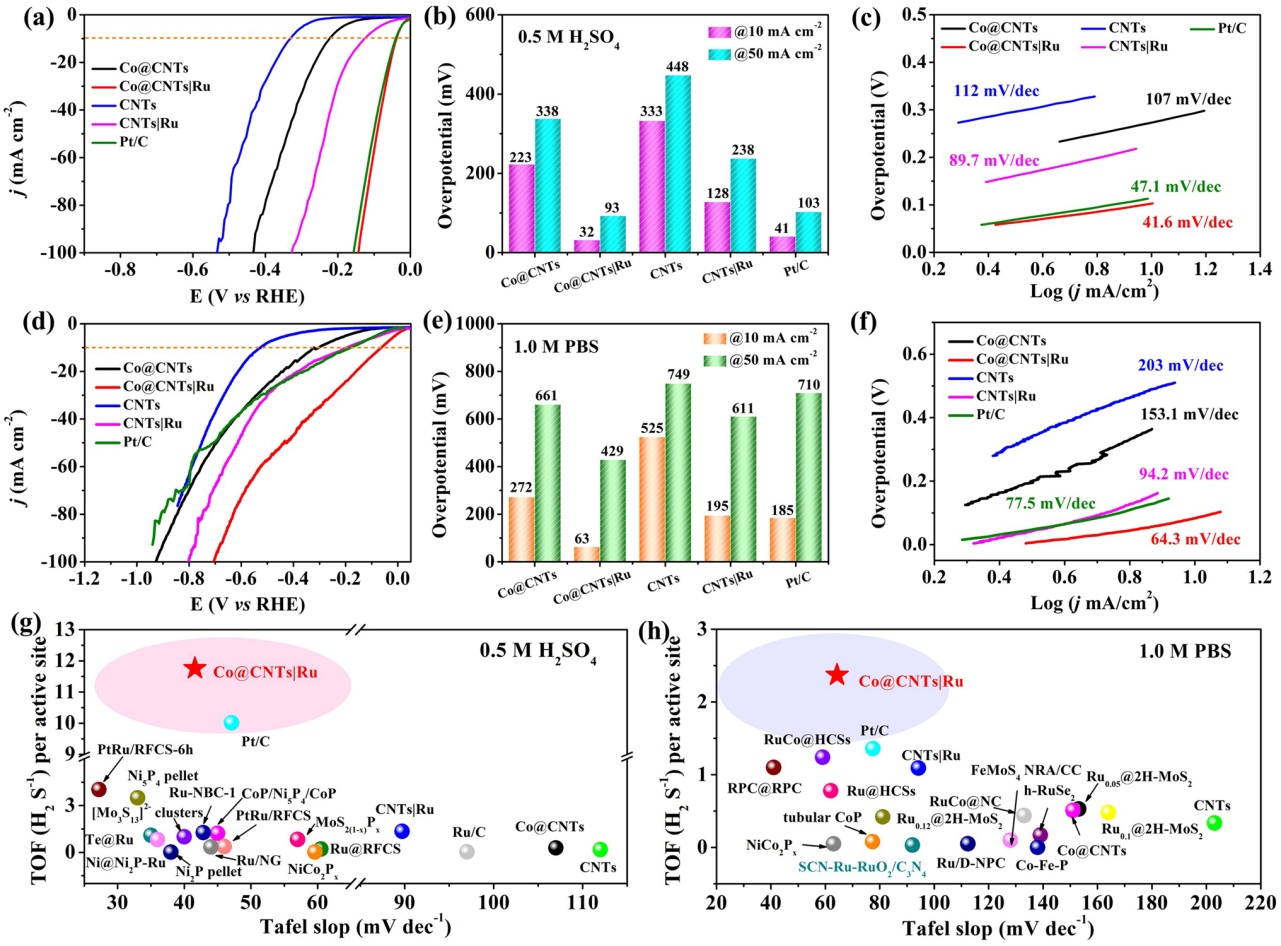
**a** LSV curves, **b** overpotentials at a current density of 10 mA cm^−2^, **c** Tafel plots of Co@CNTs, Co@CNTs|Ru, CNTs, CNTs|Ru and Pt/C electrocatalyst in 0.5 M H_2_SO_4_ solution; **d** LSV curves, **e** overpotentials at a current density of 10 mA cm^−2^, **f** Tafel plots of Co@CNTs, Co@CNTs|Ru, CNTs, CNTs|Ru and Pt/C electrode in 1.0 M PBS solution; **g − h** TOF values of Co@CNTs, Co@CNTs|Ru, CNTs, CNTs|Ru, Pt/C catalysts and other recently reported HER electrocatalysts under a overpotential of 100 mV in 0.5 M H_2_SO_4_ and 1.0 PBS electrolytes

An ideal electrocatalyst is expected to work effectively over a wide pH range to satisfy the different applications [43]. Therefore, the HER performance of Co@CNTs|Ru catalyst was also examined in 0.5 M H_2_SO_4_ and 1.0 M PBS electrolytes, respectively. As shown in Fig. 5a-b, Co@CNTs|Ru catalyst still exhibited the lowest overpotential of 32 mV at a current density of 10 mA cm^−2^ among the investigated catalyst in the acidic solution (Table S5). Similarly, the Co@CNTs|Ru catalyst also exhibited an excellent electrocatalytic activity in 1.0 PBS (Fig. 5d). As expected, Co@CNTs|Ru could achieve 63 mV@10 mA cm^−2^, much better than those of Co@CNTs (272 mV@10 mA cm^−2^), CNTs (525 mV@10 mA cm^−2^), CNTs|Ru (195 mV@10 mA cm^−2^), and commercial 20% Pt/C (185 mV@10 mA cm^−2^) and all other reported catalysts (Fig. 5e and Table S6). The exceptional catalytic activity of Co@CNTs|Ru catalyst was also demonstrated by the lowest Tafel slope values 41.6 and 64.3 mV dec^−1^ in 0.5 M H_2_SO_4_ and 1.0 M PBS solution, respectively (Fig. 5c and f), in comparison to those of Co@CNTs (107 and 153.1 mV dec^−1^), CNTs (112 and 203 mV dec^−1^), CNTs|Ru (89.7 and 94.2 mV dec^−1^), and commercial 20% Pt/C (47.1 and 77.5 mV dec^−1^). Meanwhile, the charge transfer resistance (*R*_ct_) of Co@CNTs|Ru catalyst was also much smaller than those of Co@CNTs, CNT, CNTs|Ru, and 20% Pt/C (Fig. S17, Tables S7 and S8), meaning a favorable charge transfer kinetics in both 0.5 M H_2_SO_4_ and 1.0 M PBS electrolytes.

The operation stability of Co@CNTs|Ru catalyst was also evaluated. As shown in Fig. S18, the polarization curve obtained after 5000 cycles of CV test shows negligible degradation, demonstrating the remarkable stability of Co@CNTs|Ru catalyst in both 0.5 M H_2_SO_4_ and 1.0 M PBS electrolytes, which was also verified by the chronoamperometry test (Fig. S18 inset). The TOF values of Co@CNTs|Ru were calculated to be 11.76 and 2.37 s^−1^ at η = 100 mV in 0.5 M H_2_SO_4_ and 1.0 M PBS solution, respectively (Fig. S19), higher than those of 20% Pt (10.01 and 1.35 s^−1^) and other reported electrocatalysts (Fig. 5g-h, Tables S9 and S10). All the above results further reveal that the Co@CNTs|Ru catalyst possesses an exceptional catalytic activity at all pH conditions together with a low materials cost, suggesting that it has the great potential for HER in complex environments [44].

**Fig. 6:**
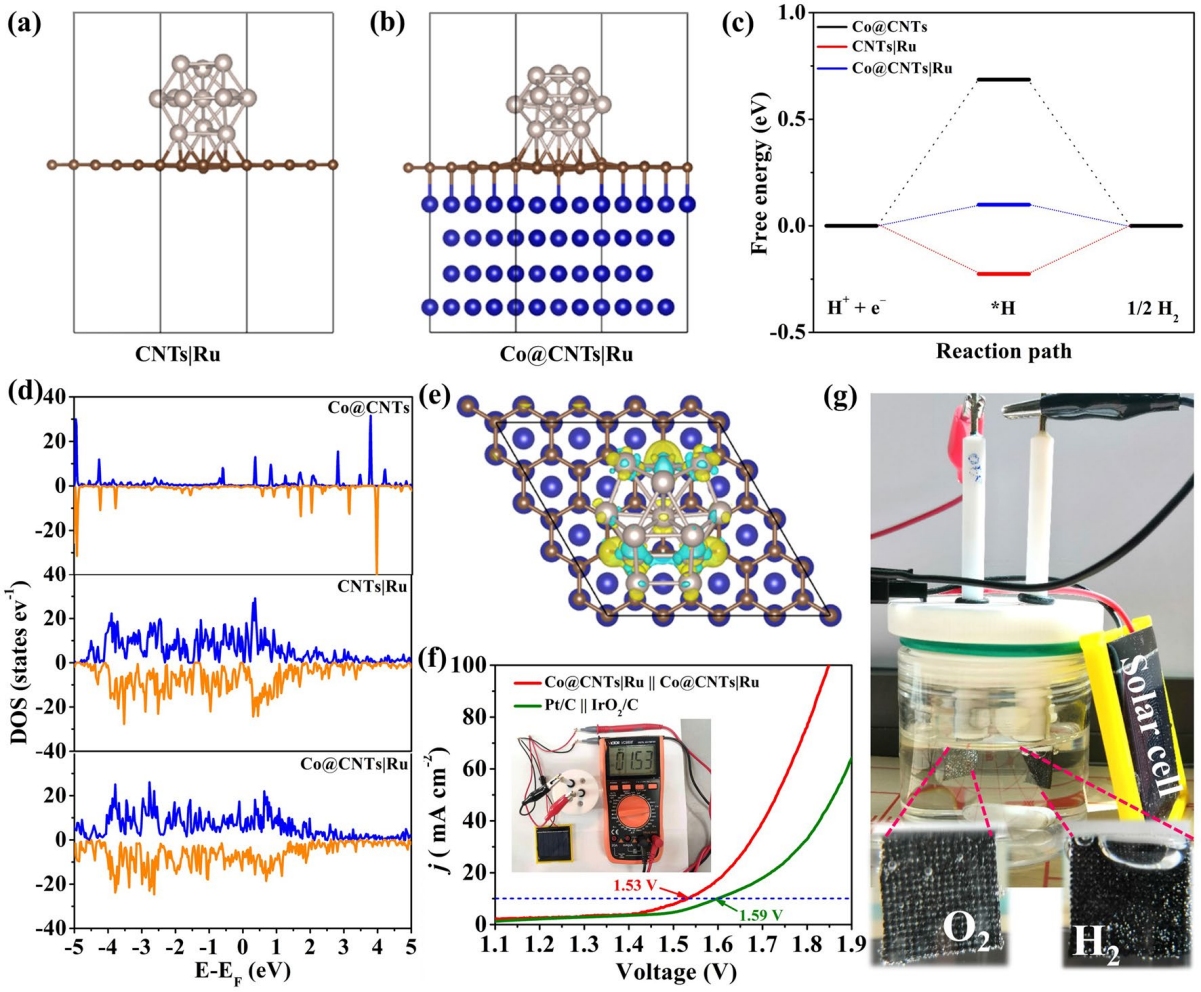
Atomistic structures of the **a** CNTs|Ru and **b** Co@CNTs|Ru in HER process; **c** corresponding free-energy diagram of Δ*G*_H*_; **d** DOS of Co@CNTs, CNTs|Ru and Co@CNTs|Ru system; **e** the charge density redistributions of Co@CNTs|Ru system (the blue, brown and white balls represent Co, C, and Ru atoms); **f** LSV curve of the typical two-electrode system by employing Co@CNTs|Ru as electrocatalysts; commercial Pt/C || IrO_2_/C was also tested for comparison (Inset: digital photograph of solar panel-assisted water splitting device); **g** the magnified photos of electrolyzer

The superior performance of Co@CNTs|Ru catalyst can be attributed to the synergistic effects of inner Co and outer Ru spatially separated by walls of CNTs. To unveil the electronic coupling effect on the HER activity, we conducted the density functional theory (DFT) calculations. For comparison, three geometric models of Co@CNTs|Ru, Co@CNTs and CNTs|Ru were constructed (Figs. 6a, b and S20). The hydrogen adsorption Gibbs free energy (Δ*G*_H*_), a widely used descriptor for HER activity [45, 46], on the surfaces of Co@CNTs|Ru, Co@CNTs and CNTs|Ru were calculated. As shown in Fig. 6c, Δ*G*_H*_ of CNTs|Ru was calculated to be − 0.246 eV, validating too strong hydrogen adsorption. In contrast, Δ*G*_H*_ of Co@CNTs|Ru was deduced to be 0.099 eV, implying the weak adsorption of hydrogen. Interestingly, upon introducing Ru onto the surface of Co@CNTs, the Δ*G*_H*_ was effectively modulated. By cooperating the Co, Ru, and CNTs, the Δ*G*_H*_ of Co@CNTs|Ru was close to zero, meaning a favorable H* adsorption and desorption [47]. As the *d*-band center is highly correlated with the metal adsorbate interaction, the density of states (DOS) of Co@CNTs|Ru, Co@CNTs and CNTs|Ru catalysts were also calculated and compared (Fig. 6d). Notably, the local states near the Fermi levels of Co@CNTs|Ru and CNTs|Ru were significantly higher than that of Co@CNTs, indicating the introduction of Ru was beneficial to tuning the electron structure of Co@CNTs|Ru. Meanwhile, the local state of Co@CNTs|Ru catalyst was slightly higher than that of CNTs|Ru, which further proved that the confined inner Co and loaded outer Ru would induce charge redistribution and synergistic electron coupling, thus optimizing the Δ*G*_H*_ [48]. To further clarify the tuning mechanism from the electronic state, the charge density difference diagrams are shown in Figs. 6e and S21-S22. Compared with the charge density difference diagrams of Co@CNTs and CNTs|Ru, Co@CNTs|Ru exhibited a stronger electron aggregation (yellow area) between the Co, CNTs, and Ru species, revealing that the introduction of Ru would lead to a strong charge transfer between Ru and Co@CNTs, altering the π-conjugated system of CNTs and creating the in-plane charge polarization in the CNTs, thus optimizing the binding strength of C with H [49].

Overall, the role of the inner and outer surfaces of CNTs for HER can be discussed as following: i) CNTs-encapsulated inner Co can effectively reduce the corrosion of Co and improve the catalytic stability. ii) The outer surface of CNTs was employed to anchor Ru nanoparticles, ensuring a uniform dispersion. iii) The inner Co and loaded outer Ru would induce charge redistribution and a synergistic electron coupling, not only optimizing the adsorption energy of H intermediates but also exposing more active sites and promoting electron/mass transfer. As a result, the Co@CNTsǀRu catalyst exhibited a superior HER catalytic performance in a pH-universal electrolysis system.

To further demonstrate the practical application of the as-developed Co@CNTs|Ru catalyst for water splitting, two-electrode configuration electrolyzer with Co@CNTs|Ru catalyst as the both cathode and anode was constructed. As displayed in Fig. 6f, the Co@CNTs|Ru could drive a current density of 10 mA cm^–2^ at an ultralow cell voltage of 1.53 V in alkaline medium, which was superior to that of Pt/C || IrO_2_/C couple (1.59 V). In addition, the chronoamperometric curve (Fig. S23) showed a negligible degradation after continuous 24 h operation, indicating the excellent durability of Co@CNTs|Ru || Co@CNTs|Ru for overall water splitting. Encouragingly, a solar-to-hydrogen system as a promising way to realize economic and sustainable H_2_ production was also assembled. The electrolyzer using the Co@CNTs|Ru || Co@CNTs|Ru catalyst could be powered by a commercial solar panel illuminated under the sunlight (inset in Fig. 6f). Accordingly, obvious H_2_ and O_2_ bubbles at the surfaces of both electrodes could be observed (Fig. 6g), demonstrating its practical feasibility in the future energy conversion application.

## Conclusion

In summary, we have developed a multicomponent electrocatalyst with ZIF-derived carbon nanotubes that encapsulate Co nanoparticles and have trace Ru nanoclusters deposited onto the outer tube walls (Co@CNTs|Ru). The CNTs-enabled spatial confinement and separation of Co and Ru would intrigue a synergistic electron coupling, leading to more exposure of active sites, a near-zero value of Δ*G*_H*_ and a rapid electron/mass transfer process. As a result, the developed Co@CNTs|Ru electrocatalyst exhibits an unprecedented HER activity with extremely low overpotentials of 10, 32, and 63 mV at 10 mA cm^−2^ in alkaline, acidic and neutral media, respectively, not only outperforming the state-of-the-art commercial Pt/C catalyst but also representing a new record among the previously reported HER catalysts. The current work may pave a new avenue to design low cost and highly active HER catalyst for applications in water splitting.

## Supplementary Information

Below is the link to the electronic supplementary material.

Supplementary file1 (PDF 1877 KB)
