# Supplementary material for: Inner Co Synergizing Outer Ru Supported on Carbon Nanotubes for Efficient pH-Universal Hydrogen Evolution Catalysis
Source: Nanomicro Lett. 2022 Sep 14;14:186. doi: 10.1007/s40820-022-00933-2 (PMC9475008; doi:10.1007/s40820-022-00933-2)
Supplement: Supplementary file 1 — Supplementary file1 (PDF 1877 KB) [file 40820_2022_933_MOESM1_ESM.pdf]

Supporting Information for

Inner Co Synergizing Outer Ru Supported on Carbon Nanotubes for Efficient pH-Universal Hydrogen Evolution Catalysis

Jian Chen[1], Yuan Ha[2], Ruirui Wang[3], Yanxia Liu[1], Hongbin Xu[3], Bin Shang[4,*], Renbing Wu[3,*], and Hongge Pan[1,5,*]

[1]Institute of Science and Technology for New Energy, Xi'an Technological University, Xi'an 710021, P. R. China

[2]School of Advanced Materials and Nanotechnology, Xidian University, Xi'an 710126, P. R. China.

[3] Department of Materials Science, Fudan University, Shanghai 200433, P. R. China

[4]State Key Laboratory of New Textile Materials and Advanced Processing Technologies, Wuhan Textile University, Wuhan 430073, P. R. China.

[5]State Key Laboratory of Silicon Materials and School of Materials Science and Engineering, Zhejiang University, Hangzhou 310027, P. R. China

*Corresponding authors. E-mail: bshang@wtu.edu.cn (Bin Shang); rbwu@fudan.edu.cn (Renbing Wu); honggepan@zju.edu.cn (Hongge Pan)

Supplementary Figures and Tables

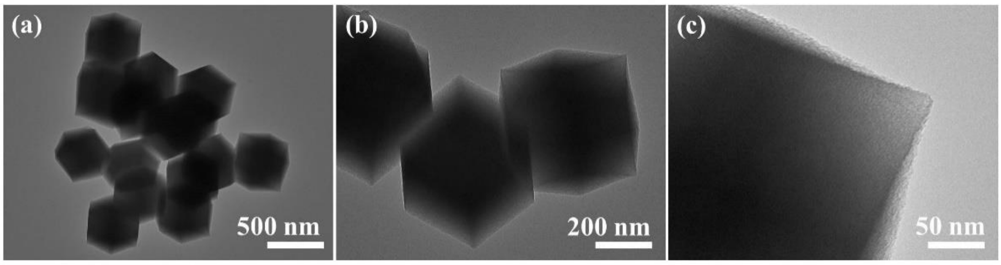

Fig. S1 TEM images of ZIF-67@Lys precursor

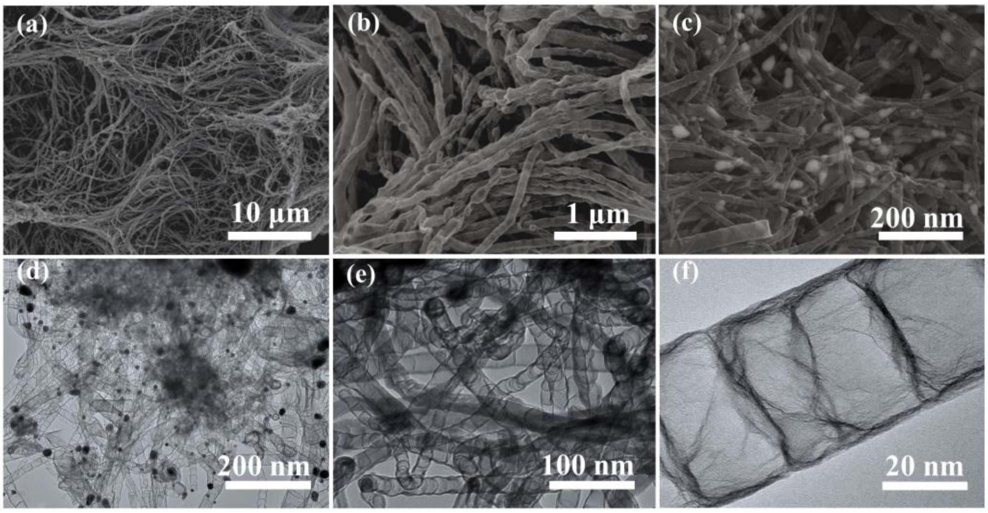

Fig. S2 a–c FESEM and **d–f** TEM images of Co@CNTs composites

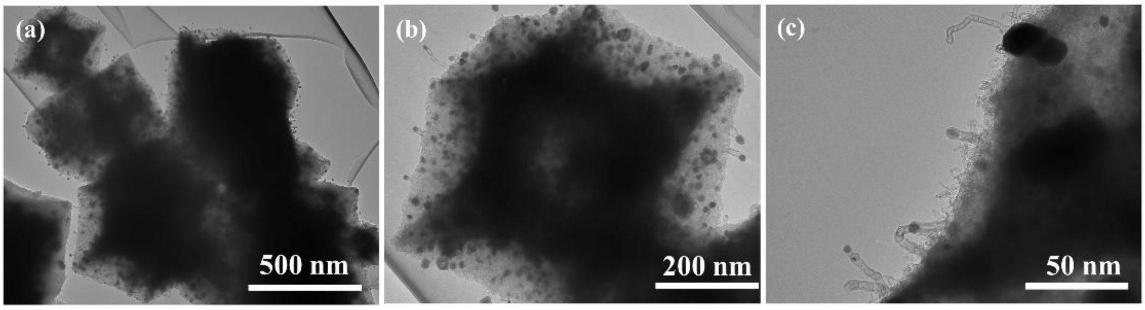

Fig. S3 TEM images of products obtained by directly pyrolyzing ZIF-67 without the introduction of Lys

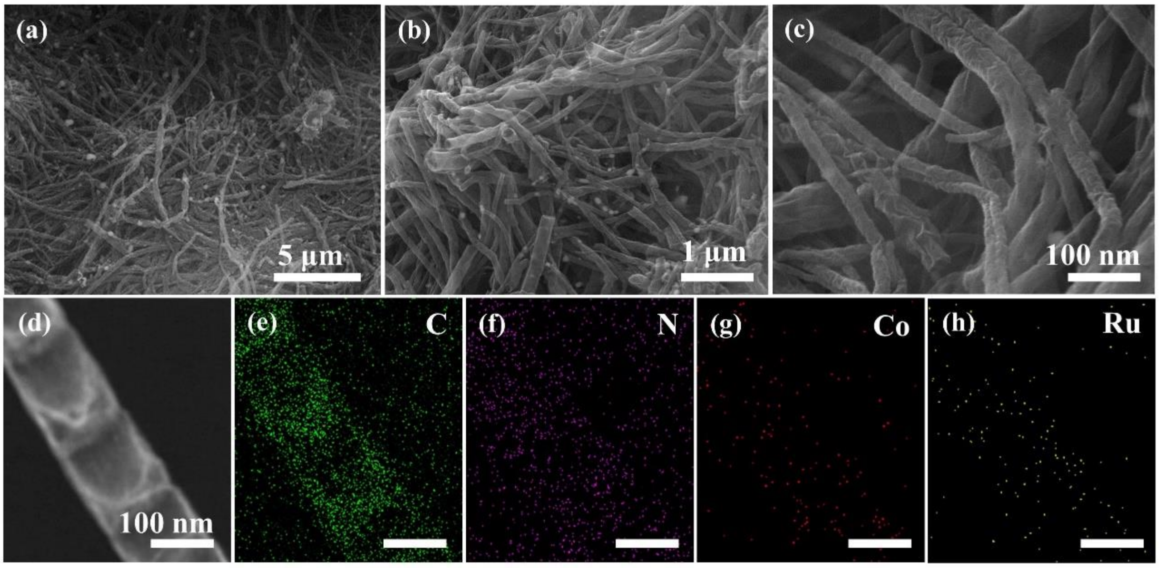

Fig. S4 a−c FESEM images of Co@CNTs|Ru composites. **d−h** HAADF-STEM images and corresponding EDX elemental mappings of selective area of Co@CNTs|Ru composites

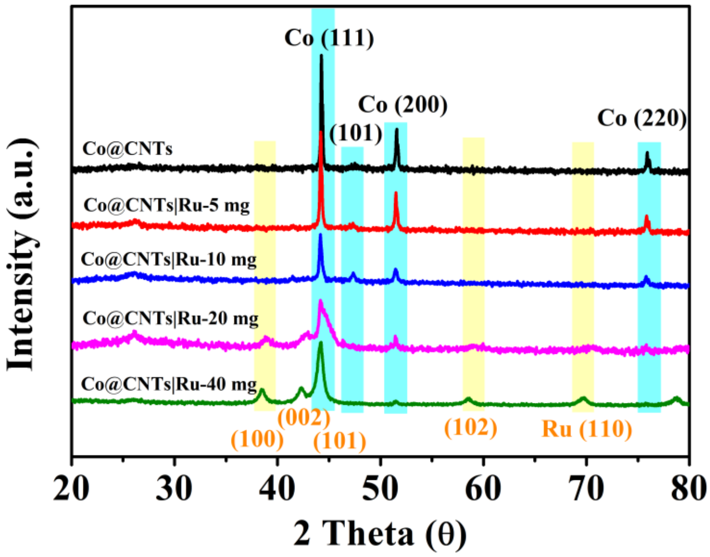

Fig. S5 XRD patterns of Co@CNTs|Ru composites with different Ru contents

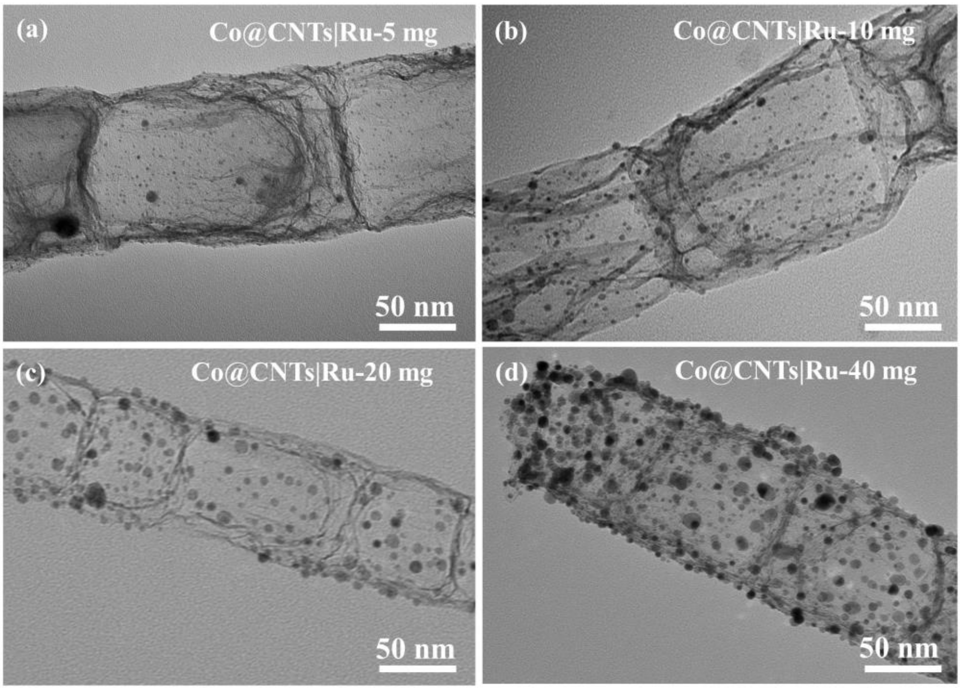

Fig. S6 TEM images of Co@CNTs|Ru composite with different Ru contents

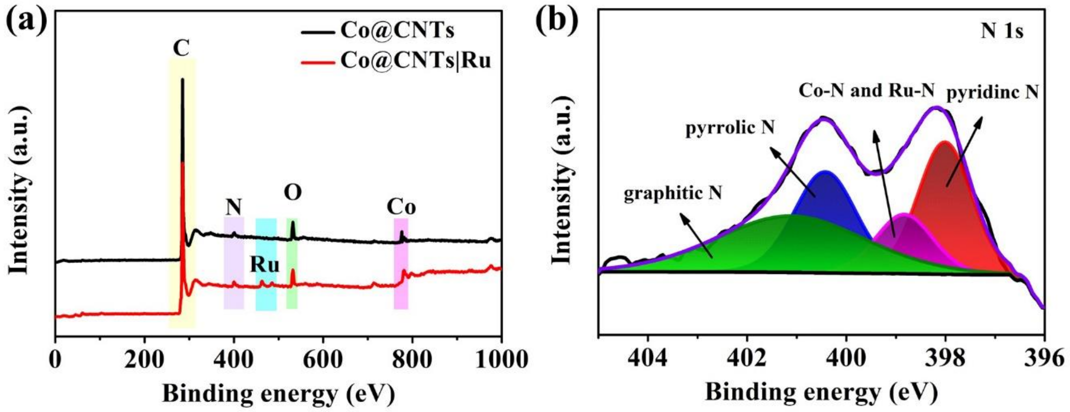

Fig. S7 **a** XPS survey spectra of Co@CNTs|Ru composites; **b** high-resolution XPS spectra of N 1s

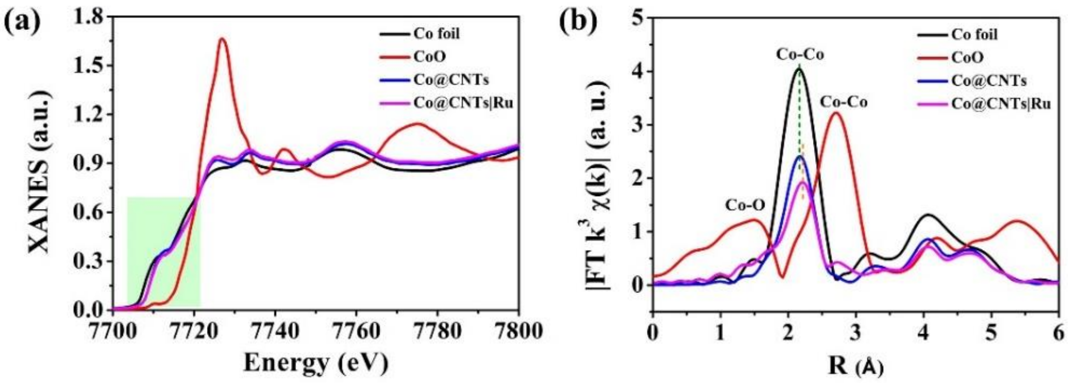

Fig. S8 Co K-edge **a** XANES and **b** EXAFS spectra of Co@CNTs|Ru and the reference samples

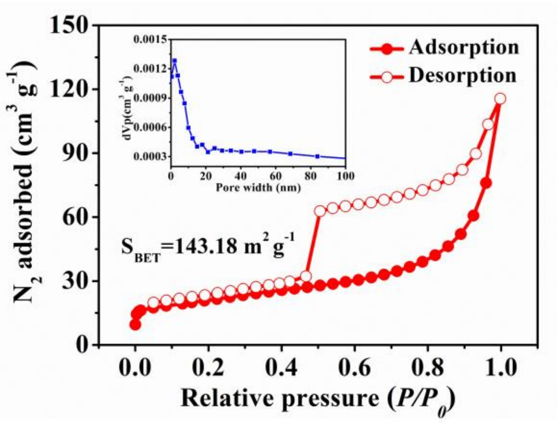

Fig. S9 N_2 sorption isotherm and desorption isotherms of Co@CNTs|Ru composites. The inset showing the pore size distribution of Co@CNTs|Ru composites

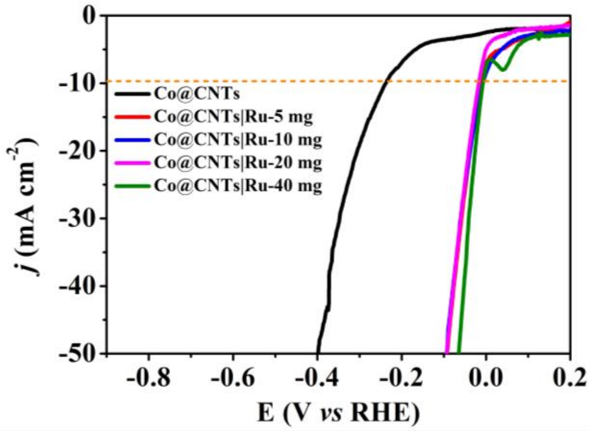

Fig. S10 LSV curves of Co@CNTs|Ru with different Ru contents in 1.0 M KOH solution

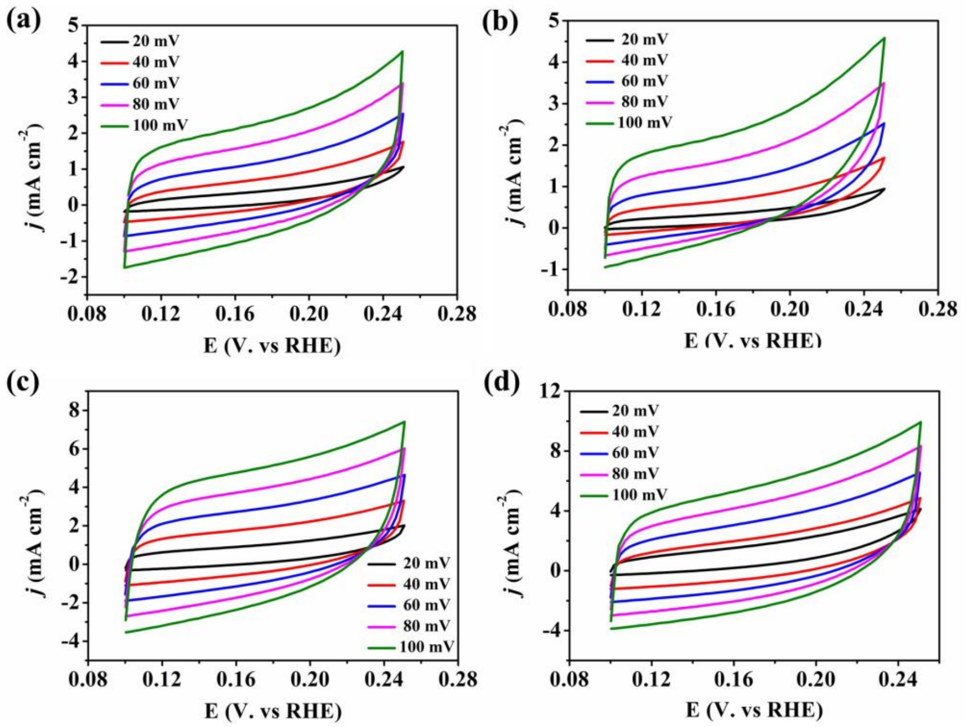

Fig. S11 CV curves for **a** Co@CNTs, **b** CNTs, **c** CNTs|Ru and **d** Pt/C electrocatalysts at different scan rates of 20, 40, 60, 80, and 100 mV s^{-1}

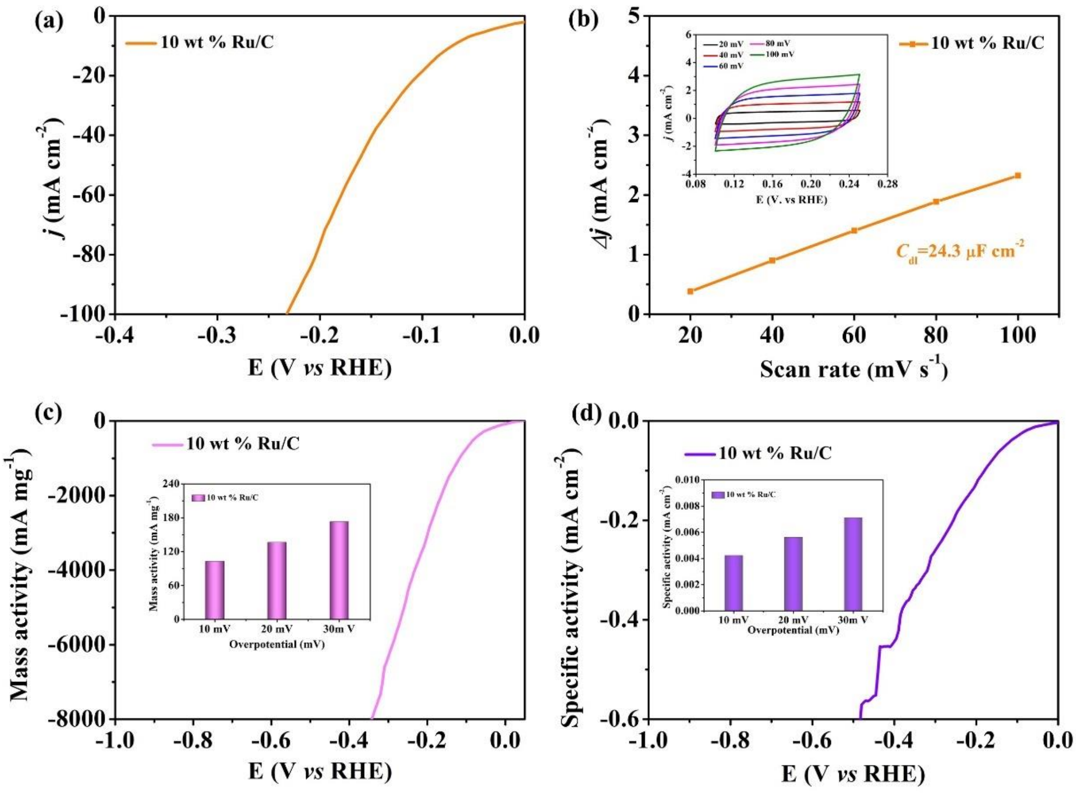

Fig. S12 a Polarization curves of 10 wt% Ru/C in N_2-saturated 1.0 M KOH solution with scan rate of 5 mV s^{-1}; **b** C_{dl} values (inset: CV curves for Ru/C electrocatalysts at different scan rates: 20, 40, 60, 80, and 100 mV s^{-1}); **c** mass activities and **d** specific activities of 10 wt% Ru/C (Inset showing the mass and the specific activities at different overpotentials of 10, 20, and 30 mV)

As shown in Fig. S12, the mass activity of 10 wt% Ru/C catalyst is around 103 mA mg^{-1}, which is much lower than that of Co@CNTs|Ru catalyst (3706 mA mg^{-1}) at an overpotential of 10 mV. The specific activity of Ru/C is 0.004 mA cm^{-2}, which is also far smaller than that of Co@CNTs|Ru (0.37 mA cm^{-2}) at an overpotential of 10 mV.

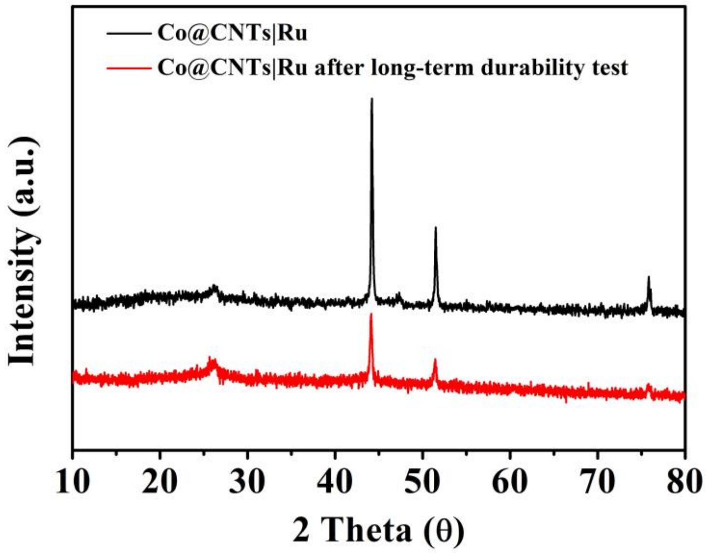

Fig. S13 XRD patterns of Co@CNTs|Ru catalyst before and after long-term durability test in 1.0 M KOH solution

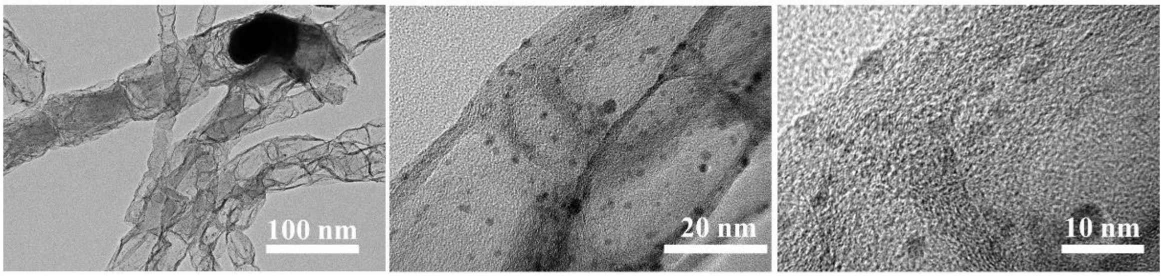

Fig. S14 TEM images of Co@CNTs|Ru after long-term durability test in 1.0 M KOH solution

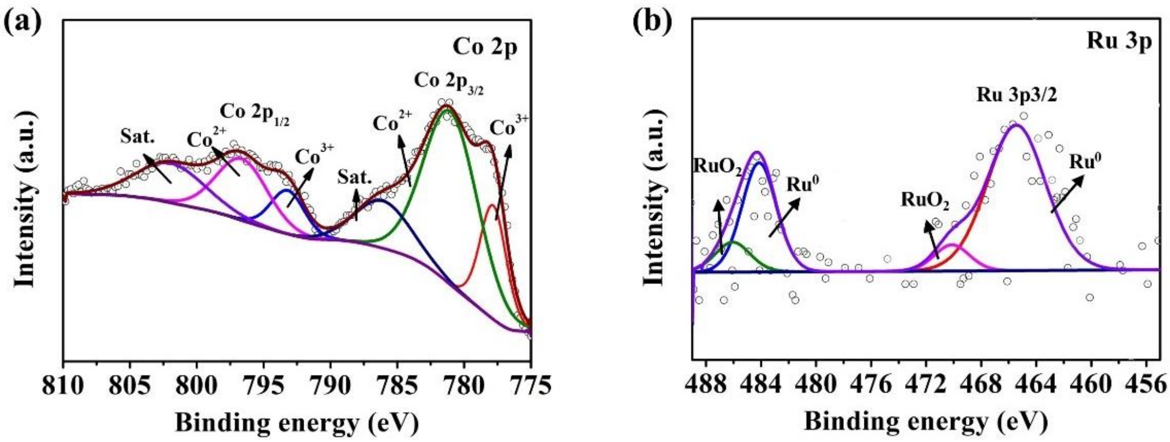

Fig. S15 High-resolution XPS spectra of **a** Co 2p and **b** Ru 3p in Co@CNTs|Ru after long-term durability test in 1.0 M KOH solution

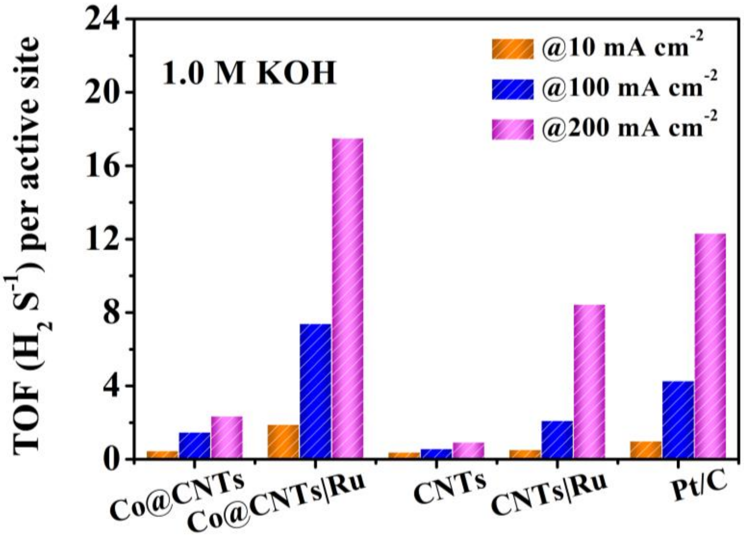

Fig. S16 TOF values of Co@CNTs, Co@CNTs|Ru, CNTs, CNTs|Ru and Pt/C catalysts at 10, 100, and 200 mV in 1.0 M KOH solution

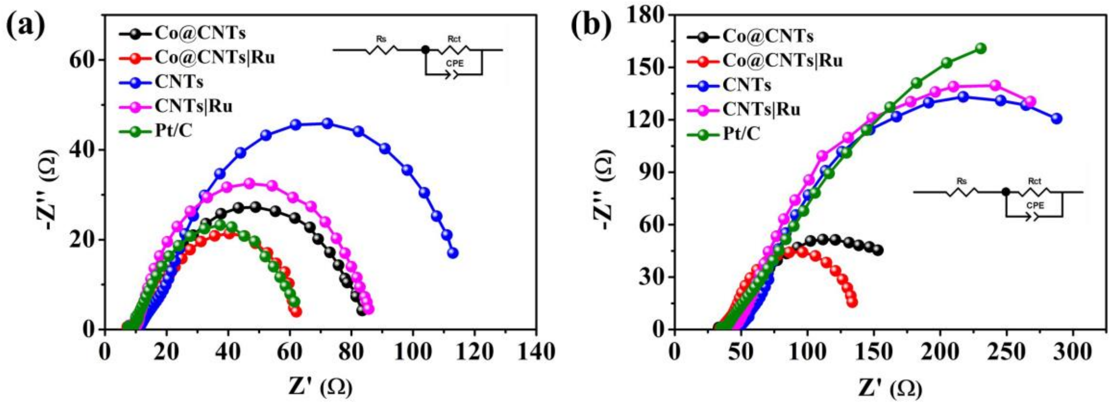

Fig. S17 EIS spectra for Co@CNTs, Co@CNTs|Ru, CNTs, CNTs|Ru and Pt/C electrocatalysts in **a** 0.5 M H_2SO_4 and **b** 1.0 M PBS solution (inset: the equivalent circuit for EIS)

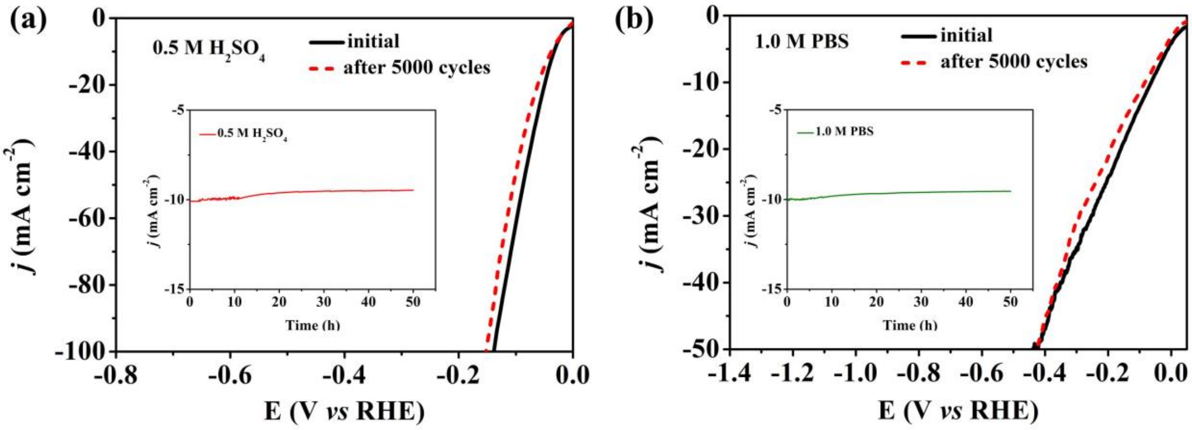

Fig. S18 Polarization curves of Co@CNTs|Ru catalyst before and after 5000 cycles of CV test in **a** 0.5 M H_2SO_4 and **b** 1.0 M PBS solution (Inset showing the *i-t* curves of Co@CNTs|Ru for 50 h)

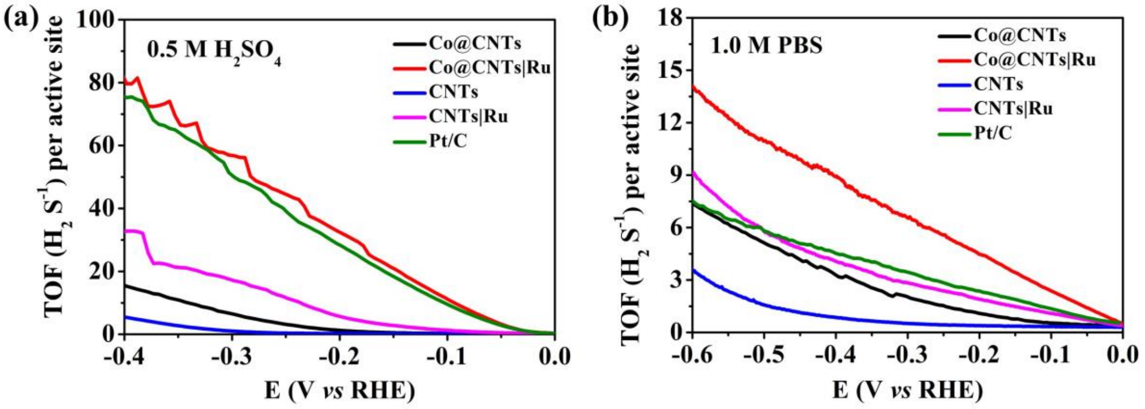

Fig. S19 TOF per surface metal site of Co@CNTs, Co@CNTs|Ru, CNTs, CNTs|Ru and Pt/C catalysts in **a** 0.5 M H_2SO_4 and **b** 1.0 M PBS electrolytes

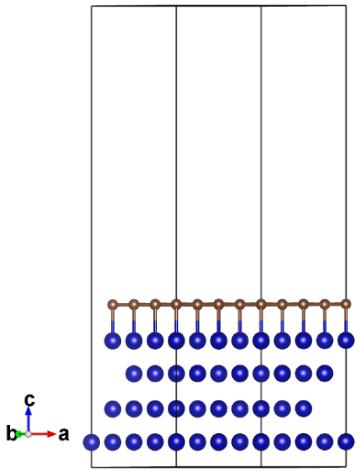

Fig. S20 Atomistic structures of the Co@CNTs in HER process

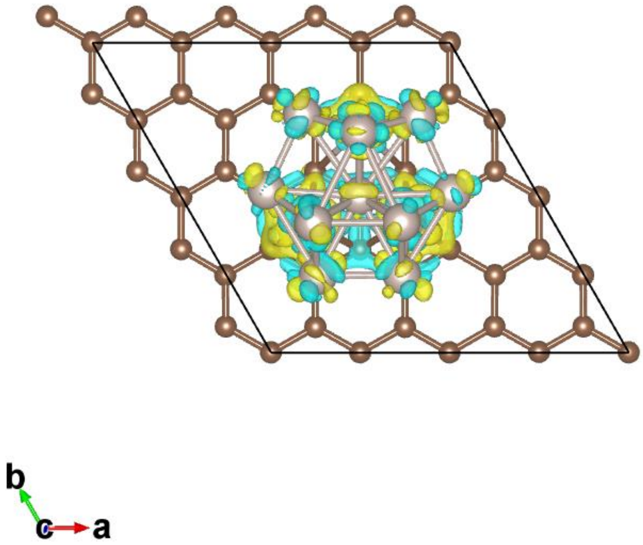

Fig. S21 Calculated charge density difference of CNTs|Ru

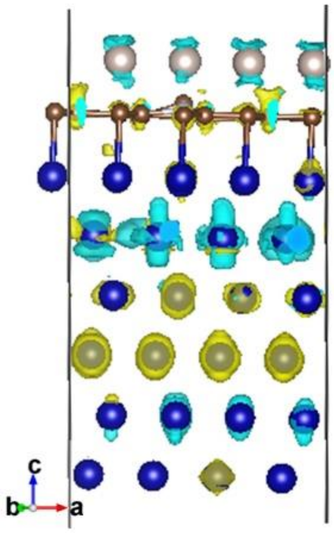

Fig. S22 Calculated charge density difference of Co@CNTs

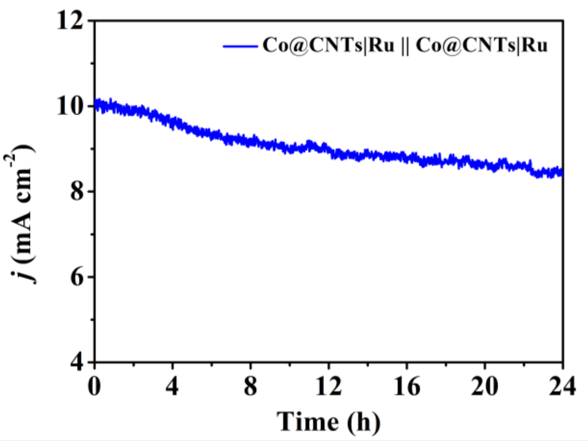

Fig. S23 Chronopotentiometry test of Co@CNTs|Ru || Co@CNTs|Ru electrolyzer at 10 mA cm^{-2}

Table S1 Co and Ru contents analysis by different methods for Co@CNTs and Co@CNTs|Ru composites

Element content	Co@CNTs	Co@CNTs|Ru	
	Co	Co	Ru
XPS (wt.%)	20.69 %	21.57 %	0.97 %
ICP-AES (wt.%)	25.64 %	25.90 %	1.04 %

Three different techniques (i.e., XPS and ICP-AES) were employed to study the Co and Ru content in both catalysts with and without the introduction of Ru.

Table S2 Comparison of the electrocatalytic activity for HER in 1.0 M KOH solution with other active electrocatalysts

Catalysts	η@10 mA cm^{-2} (mV)	Ru content (%)	Refs.
Co@CNTs|Ru	10	1.04 wt%	This work
Ru-NC-700	12	2.04 wt%	[S1]
Ru/OMSNNC	13	1.0 wt%	[S2]
CoRu@Co_4N	13	–	[S3]
Ru NCs/BNG	14	75.9 wt%	[S4]
Ru/3DNPC-500	15	7.53 wt%	[S5]
Ru@MWCNT	17	12.8 wt%	[S6]
Ru@C_2N	17	28.7 wt%	[S7]
0.27-RuO_2@C	20	–	[S8]
4H/fcc Ru NTs	23	–	[S9]
Ru@NC	26	2.0 wt%	[S10]
RuCo@NC	28	3.58 wt%	[S11]
Ni@Ni_2P-Ru	31	–	[S12]
Ru_1Ni_1-NCNFs	35	28.2 wt%	[S13]
Ru-NGC	37	6.55 wt%	[S14]
CN_x@Ru/MWCNT	39	8.0 wt%	[S15]
CoRu@NC	45	2.04 wt%	[S16]
ah-RuO_2@C	63	–	[S17]
RuP_x@NPC	74	–	[S18]
Ru/C_3N_4/C	79	–	[S19]

Table S3 EIS calculation parameters of Co@CNTs, Co@CNTs|Ru, CNTs, CNTs|Ru and 20% Pt/C electrode for HER in 1.0 M KOH solution

Sample	R_s (Ω)	Error (%)	R_{ct} (Ω)	Error (%)	CPE	Error (%)
Co@CNTs	9.22	0.79828	72.66	2.6038	0.796	1.0529
Co@CNTs|Ru	8.76	0.65447	24.51	1.72	0.925	0.425
CNTs	8.89	0.7032	96.38	1.2254	0.861	0.4421
CNTs|Ru	8.62	1.0246	81.77	2.11	0.948	0.61498
20% Pt/C	9.10	0.9002	17.91	0.72575	0.582	0.10836

Table S4 Comparison of TOFs achieved by recently reported representative HER catalysts at 100 mV overpotential in 1.0 M KOH solution

Catalysts	Tafel (mV dec^{-1})	TOF (S^{-1})	Refs.
Co@CNTs	119.7	1.48	This work
Co@CNTs|Ru	37.8	7.40	This work
CNTs	225.3	0.583	This work
CNTs|Ru	62.1	2.10	This work
Pt/C	45.3	4.27	This work
Co-NiS_2 NSs	43	0.55	*Angew. Chem. Int. Ed.*, **2019**, *58*, 18676
CoP/Ni_5P_4/CoP	43	1.22	*Energy Environ. Sci.*, **2018**, *11*, 2246
Ni_5P_4 pellet	98	0.79	*Energy Environ. Sci.*, **2018**, *11*, 2246
Ni_2P pellet	118	0.04	*Energy Environ. Sci.*, **2018**, *11*, 2246
Ni_2P	80	3.6	*Energy Environ. Sci.*, **2018**, *11*, 2246
NiMo NPs	132	0.05	*J. Am. Chem. Soc.*, **2013**, *135*, 9267
np-$Cu_{53}Ru_{47}$	35	1.139	*ACS Energy Lett.*, **2020**, *5*, 192
Ru@GnP	30	0.145	*Adv. Mater.*, **2018**, *30*, 1803676
RhO_2 clusters	30	4.2	*Adv. Mater.*, **2020**, *32*, 1908521
Ru-NBC	36.19	1.12	*Appl. Catal. B Environ.*, **2021**, *285*, 1197
Ru/OMSNNC	40.41	5.9	*Adv. Mater.*, **2021**, *33*, 2006965
RuNi/CQDs	45	5.03	*Angew. Chem. Int. Ed.*, **2020**, *59*, 1718
Ru/Co@OG	22.8	6.2	*Angew.Chem.*, **2021**, *133*, 16180
HP-Ru/C	29	5.33	*Appl. Catal. B Environ.*, **2021**, *294*, 1202
P-Ru-CoNi	69	3.1	*Small*, **2022**, *18*, 2104323
Sr_2RuO_4	51	0.9	*Nat. Commun.*, **2019**, *10*, 149
$NiCo_2Px$	34.4	0.056	*Adv. Mater.*, **2017**, *29*, 1605502
Ni-MoS_2	60	0.08	*Energy Environ. Sci.*, **2016**, *9*, 2789

Table S5 Comparison of the electrocatalytic activity for HER in 0.5 M H_2SO_4 solution with other active electrocatalysts

Catalysts	η@10 mA cm^{-2} (mV)	Ru content (%)	Refs.
Co@CNTs|Ru	32	1.04 wt%	This work
Ru-GLC	35	62.0 wt%	[S19]
RuP_2@NPC	38	23.3 wt%	[S20]
Ru_0/TiO_2	41	1.20 wt%	[S21]
Ru/CeO_2	47	–	[S22]
NiRu@N-C	50	1.86 wt%	[S23]
Ni@Ni_2P-Ru	51	–	[S12]
Ru/NG-750	53	–	[S24]
RuNi/CQDs	58	1.42 wt%	[S25]
Ru-RuO_2/CNT	63	20.4 wt%	[S26]
s-RuS_2/S-rGO	69	–	[S27]
Ru/C_3N_4/C	70	–	[S28]
Ni-doped RuO_2	78	–	[S28]
W+Ru/C	85	5.6 μg cm^{-2}	[S29]
Te@Ru	86	–	[S30]
1D-RuO_2-CNx	93	–	[S31]
Ru/MoS_2/CP	96	–	[S32]

Ni@Ni_2P-Ru	99	–	[S12]
Ru@CN	126	3.18 wt%	[S33]
$Cu_{2-x}S$@Ru NPs	129	–	[S34]

Table S6 Comparison of the electrocatalytic activity for HER in 1.0 M PBS solution with other active electrocatalysts

Catalysts	η@10 mA cm^{-2} (mV)	Ru content (%)	Refs.
Co@CNTs|Ru	63	1.04 wt%	This work
Ru-Ni_2P/NF	65	–	[S35]
Ru/3DNCN	66	29 wt%	[S36]
Ru@SC-CDs	66	–	[S37]
RuCo@CD	67	7.82 wt%	[S38]
Ru/OMSNNC	70	1.0 wt%	[S2]
3D RuCu NCs	73	–	[S39]
RuP NPs	80	21.4 wt%	[S40]
RuSA–N-Ti_3C_2Tx	81	1.1 wt%	[S41]
CuRu/CB	91	–	[S42]
Ru@CN-0.16	100	3.18 wt%	[S43]
Ru-NiFeP/NF	105	0.6 wt%	[S44]
RuP_x@NPC	110	–	[S45]
Ru-MoS_2/CC	114	0.27 wt%	[S46]
h-$RuSe_2$	119	–	[S47]
RuNi@CN-700	143	0.1219 wt%	[S48]
Ru-S-Sb/antimonene	153	18.2 wt%	[S49]
$Rh_{50}Ru_{50}$@UiO-66-NH_2	177	–	[S50]
Ru/C-2	188	2.34 wt%	[S51]
RuP_2@NC	196	3.85 wt%	[S52]

Tabl S7 EIS calculation parameters of Co@CNTs, Co@CNTs|Ru, CNTs, CNTs|Ru and Pt/C electrocatalysts for HER in 0.5 M H_2SO_4

Sample	R_s (Ω)	Error (%)	R_{ct} (Ω)	Error (%)	CPE	Error (%)
Co@CNTs	8.73	0.6244	76.08	1.4934	0.7877	1.6646
Co@CNTs|Ru	7.082	0.7186	51.46	0.9938	0.9236	1.5938
CNTs	9.20	0.698	107.92	1.027	0.9872	2.2234
CNTs|Ru	8.14	0.8274	79.95	1.2531	0.7005	1.942
20% Pt/C	7.62	0.665	53.55	0.8876	0.6894	1.36

Table S8 EIS calculation parameters of Co@CNTs, Co@CNTs|Ru, CNTs, CNTs|Ru and Pt/C electrocatalysts for HER 1.0 M PBS solution

Sample	R_s (Ω)	Error (%)	R_{ct} (Ω)	Error (%)	CPE	Error (%)
Co@CNTs	37.11	1.9357	120.74	2.0772	0.962	1.0246
Co@CNTs|Ru	39.0	1.4733	90.28	1.9376	1.053	1.0529
CNTs	45.6	2.3241	402.16	3.40	0.738	1.5547
CNTs|Ru	40.23	2.0231	362.09	2.9002	1.509	1.72
20% Pt/C	35.07	1.5622	494.55	2.88	0.869	0.79828

Table S9 Comparison of TOFs achieved by recently reported representative HER catalysts at 100 mV overpotential in 0.5 M H_2SO_4 solution

Catalysts	Tafel (mVdec^{-1})	TOF (S^{-1})	Refs.
Co@CNTs	107	0.288	This work
Co@CNTs|Ru	41.6	11.76	This work
CNTs	112	0.178	This work
CNTs|Ru	89.17	1.352	This work
Pt/C	47.1	10.013	This work
CoP/Ni_5P_4/CoP	45	1.22	*Energy Environ. Sci.*, **2018**, *11*, 2246–2252

$Ni@Ni_2P$-Ru	35	1.1	*J. Am. Chem. Soc.*, **2018**, *140*, 2731–2734
Ni_5P_4 pellet	33	3.5	*Energy Environ. Sci.*, **2015**, *8*, 1027–1034
Ni_2P pellet	38	0.015	*Energy Environ. Sci.*, **2015**, *8*, 1027–1034
PtRu/RFCS-6h	27.2	4.03	*Energy Environ. Sci.*, **2018**, *11*, 1232–1239
Ru@RFCS	60.5	0.215	*Energy Environ. Sci.*, **2018**, *11*, 1232–1239
PtRu/RFCS	46	0.375	*Energy Environ. Sci.*, **2018**, *11*, 1232–1239
Ru-NBC-1	42.84	1.27	*Appl. Catal. B Environ.*, **2021**, *285*, 1197
$NiCo_2PX$	59.6	0.021	*Adv. Mater.*, **2017**, *29*, 1605502
$[Mo_3S_{13}]^{2-}$	40	1	*Nat. Chem.*, **2014**, *6*, 248–253
$MoS_{2(1-x)}P_x$	57	0.83	*Adv. Mater.*, **2015**, *28*, 1427
Te@Ru-0.6/C	36	0.82	*Chem. Commun.*, **2019**, *55*, 1490–1493
Ru/C	97	0.036	*Chem. Commun.*, **2019**, *55*, 1490–1493
Ru/NG	44	0.35	*ACS Appl. Mater. Interfaces*, **2017**, *9*, 4, 3785–3791

Table S10 Comparison of TOFs achieved by recently reported representative HER catalysts at 100 mV overpotential in 1.0 PBS solution

Catalysts	Tafel (mV dec^{-1})	TOF (S^{-1})	Refs.
Co@CNTs	153.1	0.52946	This work
Co@CNTs|Ru	64.3	2.37076	This work
CNTs	203	0.33603	This work
CNTs|Ru	94.2	1.09241	This work
Pt/C	77.5	1.359	This work
$Ru_{0.05}@MoS_2$	151	0.51	*Appl. Catal. B Environ.*, **2021**, *298*, 120490
$Ru_{0.10}@MoS_2$	164	0.48	*Appl. Catal. B Environ.*, **2021**, *298*, 120490
$Ru_{0.12}@MoS_2$	81.1	0.42	*Appl. Catal. B Environ.*, **2021**, *298*, 120490
Ru/D-NPC	112.4	0.052	*Appl. Catal. B Environ.*, **2022**, *306*, 121095
h-$RuSe_2$	139	0.17	*Angew. Chem.*, **2021**,*133*, 7089–7093
RPC@RPC	41	1.1	*Appl. Catal. B Environ.*, **2022**, *305*, 1210
Ru-RuO_2/C_3N_4	92	0.033	*Nano Energy*, **2020**, *76*, 10507
Co-Fe-P	138	0.0013	*Nano Energy*, **2018**, *56*, 225
tubular CoP	77.35	0.08	*Int. J. Hydrog. Energy*, **2022**, *47*, 181
RuCo@HCSs	59	1.24	*ACS Sustainable Chem. Eng.*, **2019**, *7*, 18744
Ru@HCSs	62	0.78	*ACS Sustainable Chem. Eng.*, **2019**, *7*, 18744
$FeMoS_4$	128	0.1	*Chem. Commun.*, **2017**, *53*, 9000
RuCo@NC	133	0.44	*Electrochimi. Acta*, **2019**, *327*, 134985
$NiCo_2P_x$	63	0.05	*Adv. Mater.*, **2017**, *29*, 1605502

Supplementary References

[S1] B.Z. Lu, L. Guo, F. Wu, Y. Peng, J.E. Lu et al., Ruthenium atomically dispersed in carbon outperforms platinum toward hydrogen evolution in alkaline media. Nat. Commun. **10**, 631 (2019). https://doi.org/10.1038/s41467-019-08419-3

[S2] Y.L .Wu, X.F. Li, Y.S. Wei, Z.M. Fu, W.B. Wei et al., Ordered macroporous superstructure of nitrogen-doped nanoporous carbon implanted with ultrafine Ru nanoclusters for efficient pH-universal hydrogen evolution reaction. Adv. Mater. **33**(12), 2006965 (2021). https://doi.org/10.1002/adma.202006965

[S3] M.L. Zhang, J.L. Wang, Y.Q. Zhang, L. Ye, Y.Q. Gong, Ultrafine CoRu alloy nanoparticles in situ embedded in Co_4N porous nanosheets as high-efficient hydrogen evolution electrocatalysts. Dalton Trans. **50**(8), 2973–2980 (2021). https://doi.org/10.1039/D0DT04248J

[S4] S.H. Ye, F.Y. Luo, T.T. Xu, P.Y. Zhang, H.D. Shi et al., Boosting the alkaline hydrogen evolution of Ru nanoclusters anchored on B/N-doped graphene by accelerating water dissociation. Nano Energy **68**, 104301 (2020).

https://doi.org/10.1016/j.nanoen.2019.104301

[S5] Y.L. Cao, H.P. Zhang, K.K. Liu, Q.Y. Zhang, K.J. Chen, Biowaste-derived bimetallic Ru-MoO_x catalyst for the direct hydrogenation of furfural to tetrahydrofurfuryl alcohol. ACS Sustain. Chem. Eng. **7**(15), 12858–12866 (2019). https://doi.org/10.1021/acssuschemeng.9b01765

[S6] D.H. Kweon, M.S. Okyay, S.J. Kim, J.P. Jeon, H.J. Noh et al., Ruthenium anchored on carbon nanotube electrocatalyst for hydrogen production with enhanced faradaic efficiency. Nat. Commun. **11**,1278 (2020). https://doi.org/10.1038/s41467-020-15069-3

[S7] J. Mahmood, F. Li, S.M. Jung, M.S. Okyay, I. Ahmad et al., An efficient and pH-universal ruthenium-based catalyst for the hydrogen evolution reaction. Nat. Nanotechnol. **12**(5), 441–446 (2017). https://doi.org/10.1038/nnano.2016.304

[S8] H.S. Park, J.C. Yang, M.K. Cho, Y.D. Lee, S.H. Cho et al., RuO_2 nanocluster as a 4-in-1 electrocatalyst for hydrogen and oxygen electrochemistry. Nano Energy **55**, 49–58 (2019). https://doi.org/10.1016/j.nanoen.2018.10.017

[S9] Q.P. Lu, A.L. Wang, H.F. Cheng, Y. Gong, Q.B. Yun et al., Synthesis of hierarchical 4H/fcc Ru nanotubes for highly efficient hydrogen evolution in alkaline media. Small **14**(30), 1801090 (2018). https://doi.org/10.1002/smll.201801090

[S10] Z.L. Wang, K.J. Sun, J. Henzie, X.F. Hao, C.L. Li et al., Spatially confined assembly of monodisperse ruthenium nanoclusters in a hierarchically ordered carbon electrode for efficient hydrogen evolution. Angew. Chem. Int. Ed. **57**(20),5848–5852 (2018). https://doi.org/10.1002/anie.201801467

[S11] J.W. Su, Y. Yang, G.L. Xia, J.T. Chen, P. Jiang et al., Ruthenium-cobalt nanoalloys encapsulated in nitrogen-doped graphene as active electrocatalysts for producing hydrogen in alkaline media. Nat. Commun. **8**, 14969 (2017). https://doi.org/10.1038/ncomms14969

[S12] Y. Liu, S.L. Liu, Y. Wang, Q.H. Zhang, L. Gu et al., Ru modulation effects in the synthesis of unique rod-like Ni@Ni_2P-Ru heterostructures and their remarkable electrocatalytic hydrogen evolution performance. J. Am. Chem. Soc. **140**(8), 2731–2734 (2018). https://doi.org/10.1021/jacs.7b12615

[S13] M.X. Li, H.Y. Wang, W.D. Zhu, W.M. Li, C. Wang et al., RuNi nanoparticles embedded in n-doped carbon nanofibers as a robust bifunctional catalyst for efficient overall water splitting. Adv. Sci. **7**(2), 1901833 (2020). https://doi.org/10.1002/advs.201901833

[S14] Q. Song, X.Z. Qiao, L.Z. Liu, Z.J. Xue, C.H. Huang et al., Ruthenium@N-doped graphite carbon derived from carbon foam for efficient hydrogen evolution reaction. Chem. Commun. **55**(7), 965–968 (2019). https://doi.org/10.1039/C8CC09624D

[S15] W.Y. Gou, J.Y. Li, W. Gao, Z.M. Xia, S. Zhang et al., Downshifted d-band center of Ru/MWCNTs by turbostratic carbon nitride for efficient and robust hydrogen evolution in alkali. Chemcatchem **11**(7), 1970–1976 (2019). https://doi.org/10.1002/cctc.201900006

[S16] Y. Xu, Y.H. Li, S.L. Yin, H.J. Yu, H.R. Xue et al., Ultrathin nitrogen-doped graphitized carbon shell encapsulating CoRu bimetallic nanoparticles for enhanced electrocatalytic hydrogen evolution. Nanotechnology **29**(22), 225403 (2018). https://doi.org/10.1088/1361-6528/aab6c1

[S17] J.Q. Chi, W.K. Gao, J.H. Lin, B. Dong, K.L. Yan et al., Hydrogen evolution activity

of ruthenium phosphides encapsulated in nitrogen- and phosphorous-co doped hollow carbon nanospheres. ChemSuschem **11**(4), 743–752 (2018). https://doi.org/10.1002/cssc.201702010

[S18] Y. Zheng, Y. Jiao, Y.H. Zhu, L.H. Li, Y. Han et al., High electrocatalytic hydrogen evolution activity of an anomalous ruthenium catalyst. J. Am. Chem. Soc. **138**(49), 16174–16181 (2016). https://doi.org/10.1021/jacs.6b11291

[S19] Z. Chen, J.F. Lu, Y.J. Ai, Y.F. Ji, T. Adschiri et al., Ruthenium/graphene-like layered carbon composite as an efficient hydrogen evolution reaction electrocatalyst. ACS Appl. Mater. Interfaces **8**(51), 35132–35137 (2016). https://doi.org/10.1021/acsami.6b09331

[S20] Z.H. Pu, I.S. Amiinu, Z.K. Kou, W.Q. Li, S.C. Mu, RuP_2-based catalysts with platinum-like activity and higher durability for the hydrogen evolution reaction at all pH values. Angew. Chem. Int. Ed. **56**(38), 11559–11564 (2017). https://doi.org/10.1002/anie.201704911

[S21] E. Demir, S. Akbayrak, A.M. Önal, S. Özkar, Titania, zirconia and hafnia supported ruthenium (0) nanoparticles: highly active hydrogen evolution catalysts. J. Colloid Interface Sci. **531**, 570–577 (2018). https://doi.org/10.1016/j.jcis.2018.07.085

[S22] T.T. Liu, S. Wang, Q.J. Zhang, L. Chen, W.H. Hu et al., Ultrasmall Ru_2P nanoparticles on graphene: a highly efficient hydrogen evolution reaction electrocatalyst in both acidic and alkaline media. Chem. Commun. **54**, 3343–3346 (2018). https://doi.org/10.1039/C8CC01166D

[S23] Y. Xu, S.L. Yin, C.J. Li, K. Deng, H.R. Xue et al., Low-ruthenium-content NiRu nanoalloys encapsulated in nitrogen-doped carbon as highly efficient and pH-universal electrocatalysts for the hydrogen evolution reaction. J. Mater. Chem. A **6**(4), 1376–1381 (2018). https://doi.org/10.1039/C7TA09939H

[S24] R.Q. Ye, Y.Y. Liu, Z.W. Peng, T. Wang, A.S. Jalilov et al., High performance electrocatalytic reaction of hydrogen and oxygen on ruthenium nanoclusters. ACS Appl. Mater. Interfaces **9**(4), 3785–3791 (2017). https://doi.org/10.1021/acsami.6b15725

[S25] Y. Liu, X. Li, Q.H. Zhang, W.D. Li, Y. Xie et al., A general route to prepare low-ruthenium-content bimetallic electrocatalysts for pH-universal hydrogen evolution reaction by using carbon quantum dots. Angew. Chem. Int. Ed. **59**(4), 1718–1726 (2020). https://doi.org/10.1002/anie.201913910

[S26] M.T. Zhang, J.X. Chen, H. Li, P.W. Cai, Y. Li et al., Ru-RuO_2/CNT hybrids as high-activity pH-universal electrocatalysts for water splitting within 0.73 V in an asymmetric-electrolyte electrolyzer. Nano Energy **61**, 576–583 (2019). https://doi.org/10.1016/j.nanoen.2019.04.050

[S27] J. Yu, Y.N. Guo, S.S. Miao, M. Ni, W. Zhou et al., Spherical ruthenium disulfide-sulfur-doped graphene composite as an efficient hydrogen evolution electrocatalyst. ACS Appl. Mater. Interfaces **10**(40), 34098–34107 (2018). https://doi.org/10.1021/acsami.8b08239

[S28] J. Wang, Y.J. Ji, R.G. Yin, Y.Y. Li, Q. Shao et al., Transition metal-doped ultrathin RuO_2 networked nanowires for efficient overall water splitting across a broad pH range. J. Mater. Chem. A **7**(11), 6411–6416 (2019). https://doi.org/10.1039/C9TA00598F

[S29] U. Joshi, S. Malkhandi, Y. Ren, T.L. Tan, S.Y. Chiam et al., Ruthenium-tungsten

composite catalyst for the efficient and contamination-resistant electrochemical evolution of hydrogen. ACS Appl. Mater. Interfaces **10**(7), 6354–6360 (2018). https://doi.org/10.1021/acsami.7b17970

[S30] X.D. Yang, Z.X. Zhao, X. Yu, L.G. Feng, Electrochemical hydrogen evolution reaction boosted by constructing Ru nanoparticles assembled as a shell over semimetal Te nanorod surfaces in acid electrolyte. Chem. Commun. **55**(10), 1490–1493 (2019). https://doi.org/10.1039/C8CC09993F

[S31] T. Bhowmik, M.K. Kundu, S. Barman, Growth of one-dimensional RuO_2 nanowires on g-carbon nitride: an active and stable bifunctional electrocatalyst for hydrogen and oxygen evolution reactions at all pH values. ACS Appl. Mater. Interfaces **8**(42), 28678–28688 (2016). https://doi.org/10.1021/acsami.6b10436

[S32] J.L. Liu, Y. Zheng, D.D. Zhu, A. Vasileff, T. Ling et al., Identification of pH-dependent synergy on Ru/MoS_2 interface: a comparison of alkaline and acidic hydrogen evolution. Nanoscale **9**(43), 16616–16621 (2017). https://doi.org/10.1039/C7NR06111K

[S33] J. Wang, Z.Z. Wei, S.J. Mao, H.R. Li, Y. Wang, Highly uniform Ru nanoparticles over N-doped carbon: pH and temperature-universal hydrogen release from water reduction. Energy Environ. Sci. **11(4)**, 800–806 (2018). https://doi.org/10.1039/C7EE03345A

[S34] D.H. Yoon, J.Y. Lee, B. Seo, B.Y. Kim, H. Baik et al., Cactus-like hollow $Cu_{2-x}S$@Ru nanoplates as excellent and robust electrocatalysts for the alkaline hydrogen evolution reaction. Small **13**(29), 1700052 (2017). https://doi.org/10.1002/smll.201700052

[S35] C.B. Wei, X.M. Fan, X. Deng, L.Z. Ma, X. Zhang et al., Ruthenium doped Ni_2P nanosheet arrays for active hydrogen evolution in neutral and alkaline water. Energy Fuels **4**(4), 1883–1890 (2020). https://doi.org/10.1039/D0SE00010H

[S36] H. Li, M.T. Zhang, L.C. Yi, Y.J. Liu, K. Chen et al., Ultrafine Ru nanoparticles confined in 3D nitrogen-doped porous carbon nanosheet networks for alkali-acid Zn-H_2 hybrid battery. Appl. Catal. B **280**, 119412 (2021). https://doi.org/10.1016/j.apcatb.2020.119412

[S37] Y. Liu, Y.P. Yang, Z.K. Peng, Z.Y. Liu, Z.M. Chen et al., Self-crosslinking carbon dots loaded ruthenium dots as an efficient and super-stable hydrogen production electrocatalyst at all pH values. Nano Energy **65**, 104023 (2019). https://doi.org/10.1016/j.nanoen.2019.104023

[S38] T.L. Feng, G.T. Yu, S.Y. Tao, S.J. Zhu, R.Q. Ku et al., A highly efficient overall water splitting ruthenium-cobalt alloy electrocatalyst across a wide pH range via electronic coupling with carbon dots. J Mater. Chem. A **8**(19), 9638–9645 (2020). https://doi.org/10.1039/D0TA02496A

[S39] D. Cao, J.Y. Wang, H.X. Xu, D.J. Cheng, Growth of highly active amorphous RuCu nanosheets on Cu nanotubes for the hydrogen evolution reaction in wide pH values. Small **16**(37), 2000924 (2020). https://doi.org/10.1002/smll.202000924

[S40] J. Yu, Y.N. Guo, S.X. She, S.S. Miao, M. Ni et al., Bigger is surprisingly better: agglomerates of larger RuP nanoparticles outperform benchmark pt nanocatalysts for the hydrogen evolution reaction. Adv. Mater. **30**(39), 1800047 (2018). https://doi.org/10.1002/adma.201800047

[S41] H.G. Liu, Z. Hu, Q.L. Liu, P. Sun, Y.F. Wang et al., Single-atom Ru anchored in

nitrogen-doped MXene ($Ti_3C_2T_x$) as an efficient catalyst for the hydrogen evolution reaction at all pH values. J. Mater. Chem. A **8**(46), 24710–24717 (2020). https://doi.org/10.1039/D0TA09538A

[S42] J.K. Zhao, T. Pan, J.K. Sun, H.T. Gao, J.X. Guo, Cu-Ru nanoalloys on carbon black for efficient production of hydrogen in neutral and alkaline conditions. Mater. Lett. **262**, 127041 (2020). https://doi.org/10.1016/j.matlet.2019.127041

[S43] J. Wang, Z.Z. Wei, S.J. Mao, H.R. Li, Y. Wang, Highly uniform ru nanoparticles over N-doped carbon: pH and temperature-universal hydrogen release from water reduction. Energy Environ. Sci. **11**(4), 800–806 (2018). https://doi.org/10.1039/C7EE03345A

[S44] Y. Lin, M.L. Zhang, L.X. Zhao, L.M. Wang, D.L. Cao et al., Ru doped bimetallic phosphide derived from 2D metal organic framework as active and robust electrocatalyst for water splitting. Appl. Surf. Sci. **536**, 147952 (2021). https://doi.org/10.1016/j.apsusc.2020.147952

[S45] J.Q. Chi, W.K. Gao, J.H. Lin, B. Dong, K.L. Yan et al., Hydrogen evolution activity of ruthenium phosphides encapsulated in nitrogen-and phosphorous-Co doped hollow carbon nanospheres. ChemSusChem **11**(4), 11743–752 (2018). https://doi.org/10.1002/cssc.201702010

[S46] D. Wang, Q. Li, C. Han, Z.C. Xing, X.R. Yang, Single-atom ruthenium based catalyst for enhanced hydrogen evolution. Appl. Catal. B **249**, 24991–24997 (2019). https://doi.org/10.1016/j.apcatb.2019.02.059

[S47] Y.M. Zhao, H.J. Cong, P. Li, D. Wu, S.L. Chen et al., Hexagonal $RuSe_2$ nanosheets for highly efficient hydrogen evolution electrocatalysis. Angew. Chem. Int. Ed. **133**(13), 7089–7093 (2021). https://doi.org/10.1002/ange.202016207

[S48] W. Wang, J.Q. Peng, L.H. Yang, Q.L. Liu, Y.F. Wang et al., Preparation of highly dispersed Ru-Ni alloy nanoparticles on an N-doped carbon layer (RuNi@CN) and its application as a catalyst for the hydrogen evolution reaction in alkaline solution. Int. J. Electrochem. Sci. **15**, 11769–11778 (2020). https://doi.org/10.20964/2020.12.63

[S49] Y. Li, J.X. Chen, J.H. Huang, Y. Hou, L.C. Lei et al., Interfacial engineering of Ru-S-Sb/antimonene electrocatalysts for highly efficient electrolytic hydrogen generation in neutral electrolyte. Chemcomm **55**(73), 10884–10887 (2019). https://doi.org/10.1039/C9CC05522C

[S50] Z.Q. Ding, K. Wang, Z.Q. Mai, G.Q. He, Z. Liu et al., RhRu alloyed nanoparticles confined within metal organic frameworks for electrochemical hydrogen evolution at all pH values. Int. J. Hydrogen Energy **44**(45), 24680–24689 (2019). https://doi.org/10.1016/j.ijhydene.2019.07.244

[S51] H.X. Shi, L.B. Liu, Y.D. Shi, F. Liao, Y.Z. Li et al., Silicon monoxide assisted synthesis of Ru modified carbon nanocomposites as high mass activity electrocatalysts for hydrogen evolution. Int. J. Hydrogen Energy **44**(23), 11817–11823 (2019). https://doi.org/10.1016/j.ijhydene.2019.03.042

[S52] B.Y. Guo, X.Y. Zhang, J.Y. Xie, Y.H. Shan, R.Y. Fan et al., Ultrafine RuP_2 nanoparticles supported on nitrogen-doped carbon based on coordination effect for efficient hydrogen evolution. Int. J. Hydrogen Energy **46**(11), 7964–7973 (2021). https://doi.org/10.1016/j.ijhydene.2020.12.021
